# Comprehensive assessment of machine learning methods for diagnosing gastrointestinal diseases through whole metagenome sequencing data

**DOI:** 10.1080/19490976.2024.2375679

**Published:** 2024-07-07

**Authors:** Sungho Lee, Insuk Lee

**Affiliations:** a Department of Biotechnology, College of Life Science and Biotechnology, Yonsei University, Seoul, Republic of Korea; b POSTECH Biotech Center, Pohang University of Science and Technology (POSTECH), Pohang, Republic of Korea

**Keywords:** Human gut microbiome, disease diagnosis, whole metagenome shotgun sequencing, machine learning, Crohn’s disease, colorectal cancer

## Abstract

The gut microbiome, linked significantly to host diseases, offers potential for disease diagnosis through machine learning (ML) pipelines. These pipelines, crucial in modeling diseases using high-dimensional microbiome data, involve selecting profile modalities, data preprocessing techniques, and classification algorithms, each impacting the model accuracy and generalizability. Despite whole metagenome shotgun sequencing (WMS) gaining popularity for human gut microbiome profiling, a consensus on the optimal methods for ML pipelines in disease diagnosis using WMS data remains elusive. Addressing this gap, we comprehensively evaluated ML methods for diagnosing Crohn’s disease and colorectal cancer, using 2,553 fecal WMS samples from 21 case-control studies. Our study uncovered crucial insights: gut-specific, species-level taxonomic features proved to be the most effective for profiling; batch correction was not consistently beneficial for model performance; compositional data transformations markedly improved the models; and while nonlinear ensemble classification algorithms typically offered superior performance, linear models with proper regularization were found to be more effective for diseases that are linearly separable based on microbiome data. An optimal ML pipeline, integrating the most effective methods, was validated for generalizability using holdout data. This research offers practical guidelines for constructing reliable disease diagnostic ML models with fecal WMS data.

## Introduction

The gut microbiome has been linked to a range of human diseases, sparking efforts to diagnose or predict illness risk through gut microbiome profiling.^1^ Gut microbiome-based disease diagnosis offers the dual benefits of being noninvasive and frequently identifying microbiome-derived components with therapeutic potential. Therefore, this approach is promising for personalized medicine, extending beyond diagnosis to facilitate remission and potentially complete recovery.^2,3^

With the growing complexity of microbiome data, characterized by thousands of features with non-linear and nonparametric relationships, machine learning (ML) approaches are emerging as a viable alternative to traditional statistical methods for diagnostic tasks. Classical techniques frequently struggle to distinguish disease-related signatures from noise in complex biological data, whereas advanced ML algorithms excel at unraveling intricate data patterns beyond human recognition, uncovering significant biological insights. The surge in the availability of labeled data, coupled with the rapid increase in computational power, has positioned ML more favorably than ever for data-driven diagnostic methodologies.

The reduction of technical and economic obstacles in high-throughput metagenome sequencing has further accelerated research efforts. Whole metagenome shotgun sequencing (WMS) significantly improves the resolution of taxonomic profiling down to the species and strain levels and facilitates functional profiling of microbial communities by directly quantifying sequenced reads. However, WMS analysis introduces new computational challenges due to increased data complexity and sparsity, complicating the task for ML models to discern valid diagnostic markers amidst a plethora of irrelevant signals. Another complicating factor is the inherent heterogeneity of healthy microbiomes and inconsistencies in experimental procedures, adding complexity to microbiome-based diagnostics.

For a model to be viable for real-world clinical application, it must demonstrate consistent performance on previously unseen datasets. This requires the profiling of microbiomes with features pertinent to human diseases and ensuring that training datasets reflect a diverse range of populations. Additionally, it is essential to adequately address any batch effects that might distort biological signals before initiating the training process and to mitigate systematic bias through appropriate data normalization and transformation. These steps are essential to establish reliable and effective diagnostic tools in clinical settings.

While the use of 16S rRNA sequencing data in ML models for gut microbiome-based disease diagnosis has been extensively evaluated,^4^ a methodological consensus for developing optimal ML models with WMS data remains limited. One study compared classification algorithms to predict infant host characteristics like age, sex, breastfeeding status, antibiotic use, country of origin, and delivery type, using diverse gut microbiome data types including co-abundance gene groups, microbial pathways, and taxonomies.^5^ However, the applicability of these findings for disease diagnosis is uncertain. Additionally, the study did not explore how data preprocessing procedures, such as batch effect correction, normalization, and transformation, impact WMS-based disease diagnosis. Consequently, there are currently no established practical guidelines for developing WMS-based disease diagnostic ML models.

In our study, we conduct a systematic evaluation of WMS-based ML methods for diagnosing diseases, focusing on Crohn’s disease (CD) and colorectal cancer (CRC). This comprehensive assessment covers various aspects: types of microbiome features, batch correction techniques, normalization and transformation methods, and different classification algorithms. We compared these methods for each element of the ML pipeline, assessing their impact on performance across diverse populations. We developed optimal ML pipelines by integrating best methods for each disease and tested their generalizability on unseen data. The findings of this study aim to provide practical guidelines for WMS-based diagnosis in human diseases.

## Results

### Overall strategy for evaluating ML methods for WMS-based disease diagnosis

**Figure 1.**
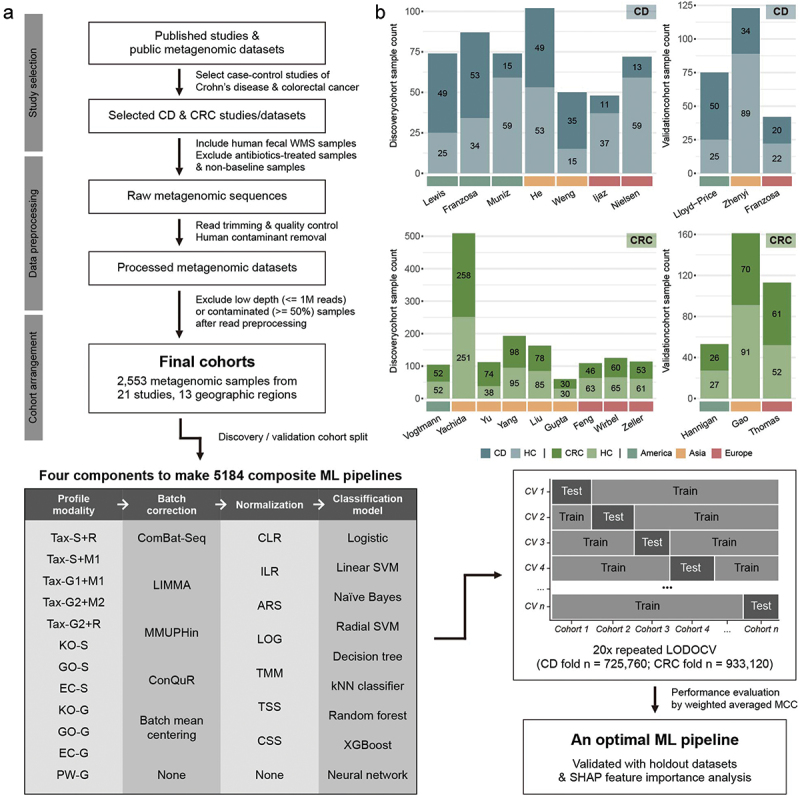
Overview of the evaluation process and statistics of the collected metagenomic datasets. (a) A flowchart of the data collection process and benchmark process. Fecal shotgun metagenome samples of eligible case-control studies for Crohn’s disease (CD) and colorectal cancer (CRC) were downloaded from public databases and checked for their quality. Only baseline samples without antibiotic treatment a month before collection were included and preprocessed consistently. After preprocessing, low-quality samples were additionally removed, and the remaining 2,553 samples were grouped by study and subsequently split into discovery and validation cohorts. We evaluated 5,184 combinations of profile modalities, batch correction methods, normalization approaches, and classification models using a 20-fold leave-one-dataset-out cross validation (LODOCV) framework, totaling 1.65 million training processes. Results were aggregated into weighted averaged unitMCC scores to identify optimal methods for each element of the ML pipeline. (b) Cohort-wise sample counts were visualized in stacked bar plots. Disease and control samples were differentiated by bar color, with the country of origin marked at the bottom, categorized into America, Asia, and Europe. The x-axis labels represent each dataset’s first author.

In this study, we gathered WMS data for CD and CRC from a variety of bibliographic databases and metagenomic sequence repositories (Figure 1a, upper left panel). The acquired WMS data underwent a standardized preprocessing protocol, during which low-quality samples with inadequate read counts or high levels of host DNA contamination were excluded. Subsequently, the 22 WMS datasets obtained (10 for CD and 12 for CRC) were divided into discovery and validation cohorts, ensuring a broad representation of geographic and ethnic diversity (Figure 1b, Supplementary table 1).

For disease diagnosis, we developed 5,184 composite ML pipelines, combining 12 distinct microbiome profiling modalities, six methods for batch correction (including the option of no correction), eight methods for data normalization and transformation (including the option of no normalization), and nine different classification algorithms (Figure 1a, lower left panel). We employed Matthew’s Correlation Coefficient (MCC)^6^ as a unified evaluation metric due to its balanced nature, which awards high scores only when a classifier performs well across all four rates of the confusion matrix.^7,8^ MCC’s class imbalance agnosticism also makes it a robust metric for real-world diagnostic scenarios. In this study, we normalized the MCC score (unitMCC) to align with the scale of the Area Under the Receiver Operating Characteristic Curve (AUROC) to enhance interpretability.

We implemented the leave-one-dataset-out cross-validation (LODOCV)^9^ approach, where all samples from a single study are held out from training and used as the test set. This method prioritizes cross-cohort generalizability and the identification of reproducible gut microbiome-based biomarkers across diverse demographic groups (Figure 1a, lower right panel). To mitigate potential biases from imbalanced class distributions between training and test data during random sampling, we performed the cross-validation analysis 20 times for each pipeline and averaged their performance scores. This resulted in a total of 725,760 evaluations for CD and 933,120 for CRC. We then summarized and ranked the methods for each element of the ML pipelines based on their unitMCC scores. The optimal ML pipeline for each disease was determined by selecting the best-performing method for each element. Finally, we assessed the overall classification performance of these optimal ML pipelines using unseen data.

### Gut-specific species-level taxonomic features provide superior profiling in WMS-based disease diagnosis

We profiled human gut microbiomes using WMS data, applying either taxonomic or functional features. We assessed seven taxonomic and five functional profile modalities, each based on a unique combination of profiling tools and reference databases (Table 1). For taxonomic profiling, we utilized genome-based tools (Kraken2 & Bracken: R) and marker-based tools (mOTUs3: M1 and MetaPhlAn4: M2), along with various reference databases (HRGM v2: S, mOTUs-DB v3.0.3: G1, ChocoPhlAn vOct22: G2). Functional profiling involved using MetaCyc pathways (PW) or gene families (Gene Ontology: GO, Enzyme Commission number: EC, KEGG Orthology: KO) as features, with their abundances measured by HUMAnN v3.6 and bowtie2, and gene family databases (HRGM v2 protein families: S, mpa_vJan21_CHOCOPhlAnSGB_202103 and uniref90_201901b_full: G). We evaluated the performance across the same set of all possible 432 base ML pipelines for each of 12 profile modalities to determine their overall impact on disease diagnosis. Additionally, we assessed the average ranking of each modality within the same 432 base pipelines, where each full pipeline consists of combinations between one of these base pipelines and one of the 12 modalities. Our analysis revealed an inverse correlation between performance scores across the 432 base pipelines and average rankings within the same 432 base pipelines, indicating consistent conclusions from both evaluations. Lastly, we examined the contribution of each profile modality to the top 1% best-performing composite ML pipelines.

**Table 1.** List of 12 distinct profile modalities for human gut microbiome.

Profile modality	Feature type	Reference database	Profiling tool
Tax-S+R	Taxonomic (Species)	HRGM v2 genomes (Gut microbiome)	Kraken2 & Bracken(Genome-based)
Tax-S+M2	Taxonomic (Species)	HRGM v2 genomes (Gut microbiome)	MetaPhlAn4 (Marker-based)
Tax-G1+M1	Taxonomic (Species)	mOTUs-DB v3.0.3 (Global microbiome)	mOTUs3 (Marker-based)
Tax-G1+R	Taxonomic (Species)	mOTUs-DB v3.0.3 (Global microbiome)	Kraken2 & Bracken(Genome-based)
Tax-G2+M2	Taxonomic (Species)	ChocoPhlAn vOct22 (Global microbiome)	MetaPhlAn4 (Marker-based)
KO-S	Functional (KEGG Orthology)	HRGM v2 protein families (Gut microbiome)	Bowtie2
EC-S	Functional (Enzyme Commission)	HRGM v2 protein families (Gut microbiome)	Bowtie2
GO-S	Functional (Gene Ontology)	HRGM v2 protein families (Gut microbiome)	Bowtie2
KO-G	Functional (KEGG Orthology)	mpa_vJan21_CHOCOPhlAnSGB_202103 and uniref90_201901b_full (Global microbiome)	HUMAnN v3.6
EC-G	Functional (Enzyme Commission)	mpa_vJan21_CHOCOPhlAnSGB_202103 and uniref90_201901b_full (Global microbiome)	HUMAnN v3.6
GO-G	Functional (Gene Ontology)	mpa_vJan21_CHOCOPhlAnSGB_202103 and uniref90_201901b_full (Global microbiome)	HUMAnN v3.6
PW-G	Functional (MetaCyc pathways)	mpa_vJan21_CHOCOPhlAnSGB_202103 and uniref90_201901b_full (Global microbiome)	HUMAnN v3.6

**Figure 2.**
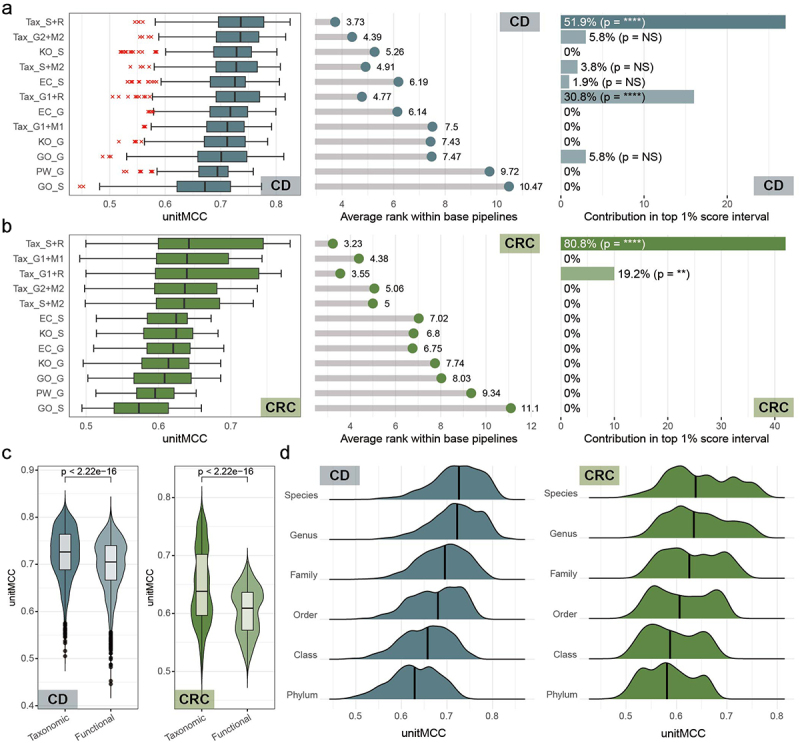
Assessment of microbiome profile modalities. (a-b) for both Crohn’s disease (CD) (a) and colorectal cancer (CRC) (b) diagnoses, distribution of unitMCC scores across 432 base pipelines and average rank within the same 432 base pipelines were plotted for each modality (left and central panel) and their contribution to the top 1% best-performing ML pipelines was shown in bar plots (right panel). Rows are ordered by descending median unitMCC. Enrichment significance in top pipelines was determined with Fisher’s exact test, where ‘NS’ denotes *p* > 0.05; ‘*’ for *p* < 0.05; ‘**’ for *p* < 0.01; ‘***’ for *p* < 0.001; and ‘****’ for *p* < 0.0001. (c-d) We conducted groupwise unitMCC comparisons. Significance was assessed using the Mann-Whitney U test. (c) Violin plots depict unitMCC distributions of ML pipelines for CD (left panel) and CRC (right panel), categorized as taxonomic or functional features. (d) Ridgeline plots illustrate unitMCC distributions across taxonomic hierarchies for CD (left panel) and CRC (right panel) diagnostic scenarios.

Our analysis revealed that the taxonomic profiles created using Kraken2 & Bracken with the HRGM human gut-specific microbial genome database (Tax-S+R) achieved the best median performance score and the highest average ranking for both CD and CRC (Figure 2a-b, left and central panels). Additionally, Tax-S+R was the most prevalent profile modality among the top 1% best-performing composite ML pipelines, being featured in 51.9% and 80.8% of the top 1% pipelines for CD and CRC diagnosis, respectively (Figure 2a-b, right panels). Notably, another profile modality with significant contribution to the top 1% pipelines, Kraken2 & Bracken in combination with the global microbial genome database mOTUs-DBv3.0.3 (Tax-G1+R), also relies on taxonomic features. Our findings also indicated that pipelines with taxonomic profiles generally ranked higher than those with functional profiles (Figure 2c). This led to the confirmation of the overall superiority of taxonomic features over functional features in diagnosing both diseases. These results align with previous research, which identified taxonomies as a more effective data type than gene groups or pathways for predicting microbiome-based host characteristics.^5^

Bacterial taxonomic features cover a hierarchical range from species to phylum. To determine which taxonomic rank offers the most effective profiles for disease diagnosis, we constructed and compared 12,960 pipelines across various taxonomic ranks. Our observations revealed a gradual enhancement in prediction performance, with species-level features yielding the best results for both diseases (Figure 2d). Collectively, these findings suggest that taxonomic features generally hold an advantage over functional features in WMS-based diagnosis of CD and CRC.

### Batch correction does not consistently improve ML models for WMS-based disease diagnosis

We evaluated batch effect correction methods potentially crucial for developing ML models for disease diagnosis using diverse, multi-cohort datasets. Effectively removing batch effects while retaining biological variance is key for discerning genuine biological signals in data from multiple batches. Recent advancements have broadened the options for batch effect correction in human microbiome data analysis, especially for metagenomics data known for its sparse and zero-inflated characteristics.^10^ We tested five batch correction methods: 1) ComBat-seq,^11^ initially designed for bulk RNA-seq data, 2) LIMMA,^12^ a versatile tool for analyzing gene expression data from microarray and RNA-seq experiments, 3) MMUPHin,^13^ tailed for covariate-controlled meta-analysis of microbial taxonomic and functional profiles, 4) ConQuR,^14^ developed specially for zero-inflated and over-dispersed microbial read count data, and 5) Naïve batch mean centering,^15^ a simple batch-wise mean centering approach that are blind to data structure and biological covariates.

**Figure 3.**
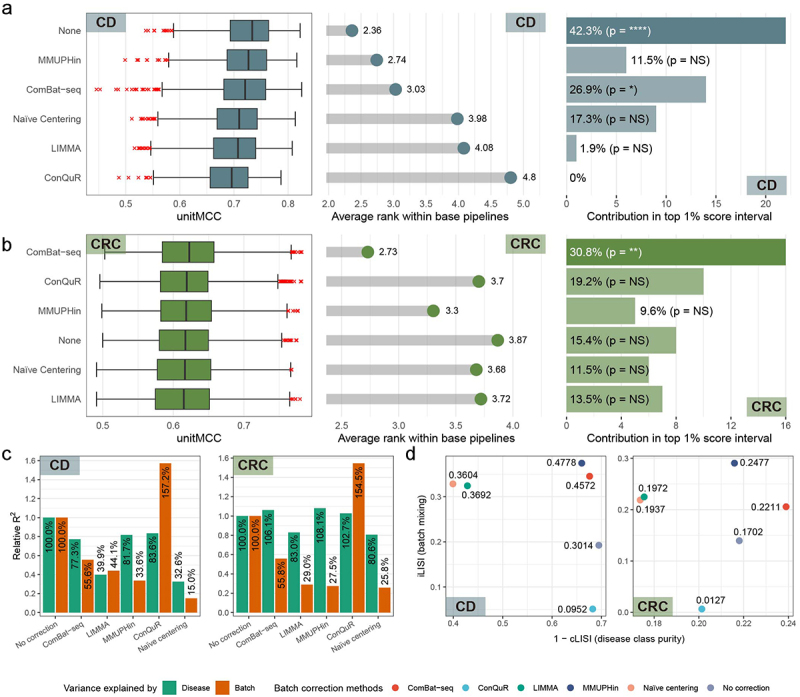
Assessment of batch correction methods. (a-b) for both Crohn’s disease (CD) (a) and colorectal cancer (CRC) (b) diagnoses, distribution of unitMCC scores across 864 base pipelines and average rank within the same 864 base pipelines were plotted for each batch correction method (left and central panel) and their contribution to the top 1% ML pipelines was shown in bar plots (right panel). Rows are ordered by descending median unitMCC. Enrichment in the top score range was analyzed with Fisher’s exact test, where ‘NS’ denotes *p* > 0.05; ‘*’ for *p* < 0.05; ‘**’ for *p* < 0.01; ‘***’ for *p* < 0.001; and ‘****’ for *p* < 0.0001. (c) The relative percentages of PERMANOVA R2 values, indicating variance explained by disease (green) or batch label (orange), are shown in bar plots for both CD (left panel) and CRC (right panel). (d) Scatterplots illustrate normalized LISI values for various batch correction methods in CD (left panel) and CRC (right panel). The x-axis represents inversed cLISI values, and the y-axis shows iLISI values, with F1^LISI^ scores labeled near each point.

In diagnosing CD, we unexpectedly found that the best prediction performance occurred with no batch correction, a trend also seen in the top 1% best-performing composite ML pipelines (Figure 3a). Surprisingly, all tested batch correction methods underperformed compared to the no correction option. In contrast, for CRC diagnosis, data corrected with ComBat-seq yielded the best predictions (Figure 3b). Notably, ComBat-seq was the only method to significantly outperform the no correction option (*p* < 0.05 by Mann-Whitney U test) and was the sole method predominant in the top 1% pipelines. The contributions of other methods, including the no correction option, were not significant. These findings suggest that batch effect correction does not uniformly enhance the performance of WMS-based disease diagnosis.

To understand why batch effect correction was not beneficial for WMS-based disease diagnosis, we analyzed the change in variance explained by batch or disease labels before and after correction using a permutational multivariate analysis of variance (PERMANOVA) test (Figure 3c), based on robust Aitchison distance^16^ between samples. For CD, all batch correction methods, except ConQuR, reduced variance due to batch information, but notably, they all failed to maintain disease label variance, possibly leading to poorer classification performance compared to no correction. In the case of CRC, methods that outperformed the no correction approach (ComBat-seq, MMUPHin, and ConQuR) enhanced variance related to the disease label. These findings highlight the importance of preserving variance associated with disease state during batch correction to improve the efficacy of WMS-based disease diagnosis in multicohort datasets.

To further evaluate batch correction methods, we used the local inverse Simpson index (LISI) score, a k-nearest neighbor graph-based local diversity metric originally developed for single-cell transcriptome dataset integration quality assessment.^17^ LISI measures the ‘degree of variety’ within local neighborhoods, which ideally should be high for batch labels (measured by integration LISI, iLISI) and low for disease labels (measured by cell-type LISI, cLISI) when batch effects are effectively removed. We calculated iLISI and cLISI values using the first 50 principal components of robust centered log-ratio transformed data, then computed the median LISI values for each batch correction method into a harmonic mean of iLISI and cLISI scores (F1^LISI^ score). Before deriving the F1^LISI^ score, we scaled iLISI and cLISI to a 0–1 range, and inverted cLISI by subtracting from 1 for simplicity. For diagnosing CD, MMUPHin had the highest F1^LISI^ score (0.4778), followed by ComBat-seq (0.4572). Conversely, for CRC diagnosis, ComBat-seq led, followed by MMUPHin. Interestingly, the method with the highest F1^LISI^ score did not correspond to the best overall performance for either disease. Yet, the method with the top 1- cLISI score, indicating disease class purity, aligned with the best overall performance for both diseases (no correction for CD and ComBat-seq for CRC) (Figure 3d). These findings underscore the importance of preserving biological covariate differences during batch correction of data for training ML models for WMS-based disease diagnosis.

### Compositional data transformations markedly improve WMS-based disease diagnosis

Microbiome profiles, even after batch effect removal, present challenges for model training and analysis interpretation due to their inherent characteristics. Firstly, metagenomic profiles are compositional, reflecting only relative abundances.^18^ This limitation stems from the finite capacity of high-throughput sequencing instruments used in metagenomic sequencing read generation, meaning sequenced read counts do not correlate directly with the absolute abundance of microbial cells. Applying count-based strategies to this compositional data can lead to erroneous conclusions. Secondly, these profiles typically feature a high number of zeros, resulting in excessive sparsity, skewness, and overdispersion.^19^ The zero-inflated nature of microbiome profiles can be attributed to both biological and technical factors. Biologically, certain microbes may be truly absent in some environments (structural zeros), while others may remain undetected due to low sequencing depth or sampling biases (technical zeros). Properly understanding these characteristics and applying suitable normalization and transformation techniques is crucial before proceeding with data analysis.

We assessed seven methods for normalizing and transforming microbiome profile data. Firstly, sequencing depth normalization techniques – total sum scaling (TSS), cumulative sum scaling (CSS),^20^ and trimmed mean of M-values (TMM)^21^ – were evaluated for their ability to correct biases arising from varying sequencing depths across samples. Secondly, we examined distribution reshaping methods, including log transformation (LOG) and arcsine square root transformation (ARS), which aim to reduce heteroskedasticity (also known as heterogeneity of variance) and reshape skewed feature distributions into more normal-like distributions. Lastly, we explored compositional transformations, such as centered log-ratio transformation (CLR)^22^ and isometric log-ratio transformation (ILR),^23^ which effectively remove compositional constraints from microbiome datasets, facilitating the use of standard analytical techniques and operations.

**Figure 4.**
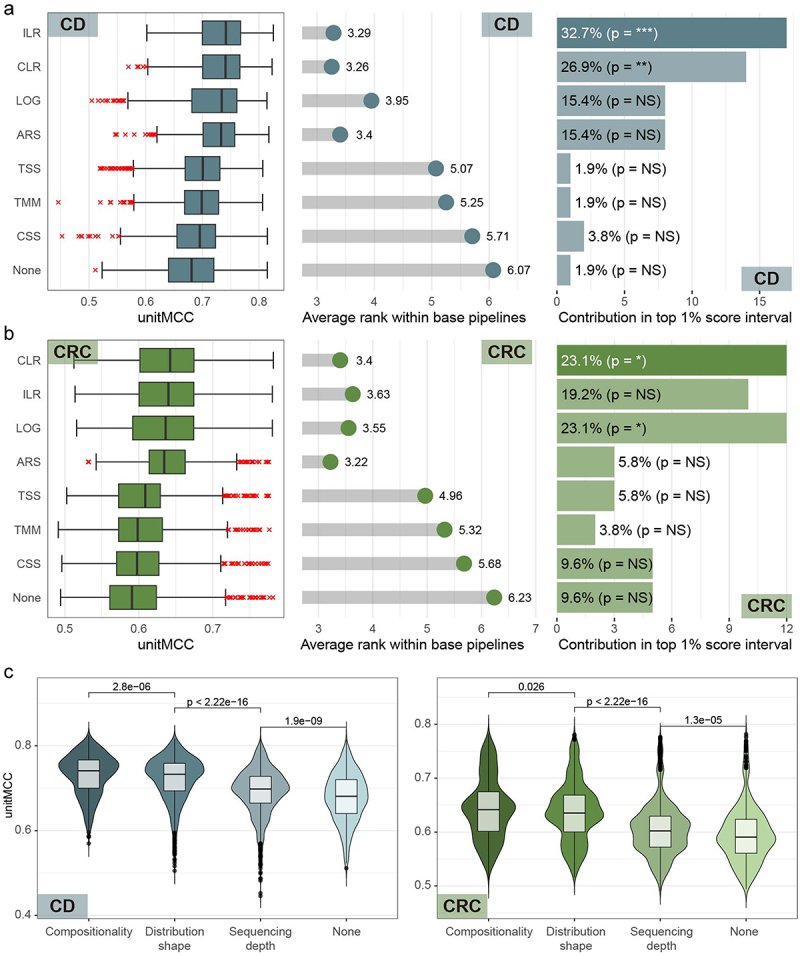
Assessment of normalization methods. (a-b) for both Crohn’s disease (CD) and colorectal cancer (CRC) diagnoses, distribution of unitMCC scores across 648 base pipelines and average rank within the same 648 base pipelines were plotted for each normalization method (left and central panel) and their contribution to the top 1% best-performing ML pipelines was shown in bar plots (right panel) in panels for CD (a) and for CRC (b). The rows in these plots are organized in descending order of median unitMCC. Enrichment significance in top pipelines was determined with Fisher’s exact test, where ‘NS’ denotes *p* > 0.05; ‘*’ for *p* < 0.05; ‘**’ for *p* < 0.01; ‘***’ for *p* < 0.001; and ‘****’ for *p* < 0.0001. (c) We displayed unitMCC distributions across all ML pipelines for CD and CRC diagnoses using violin plots, with pipelines broadly categorized into four categories of normalization approaches: compositional transformations (‘Compositionality’ – CLR and ILR), distribution reshaping methods (‘Distribution Shape’ – LOG and ARS), sequencing depth normalizations (‘Sequencing Depth’ – TSS, TMM, and CSS), and no normalization (‘None’). These plots feature CD (left panel) and CRC (right panel) diagnoses. The Mann-Whitney U test was used to assess statistical significance.

In diagnosing both diseases, all tested normalization and transformation methods enhanced classification performance compared to unprocessed data, with compositional transformations standing out significantly. For CD diagnosis, ILR and CLR transformations achieved the best results and were most common in the top 1% best-performing ML pipelines (Figure 4a). For CRC diagnosis, transformations such as CLR, ILR, LOG, and ARS yielded the best performances. However, it was CLR and LOG that emerged as the two most prevalent transformation methods within the top 1% of best-performing ML pipelines (Figure 4b). We also assessed performance across different normalization categories: compositional (CLR, ILR), distribution reshaping (LOG, ARS), and sequencing depth adjustments (TSS, CSS, TMM). This analysis reaffirmed that compositional transformations led to the highest performance in both diseases, followed by distribution reshaping, sequencing depth normalizations, and no normalization (Figure 4c). These findings suggest that merely adjusting for sequencing depth bias is inadequate for modeling ML pipelines for WMS-based disease diagnosis, and that acknowledging the compositional nature of microbiome profiles is crucial for developing optimal ML pipelines.

### Nonlinear ensemble models excel generally, yet linear models are better for linearly separable microbiome diseases

In our final phase, we assessed nine classification algorithms, each with distinct capabilities in forming decision boundaries – hypersurfaces that divide the feature space into classes. Linear models create hyperplane decision boundaries based on linear combinations of input features. We tested three such models: logistic regression (LR), naïve Bayes classifier (NBC), and linear support vector machine (Linear SVM). For capturing non-linear relationships between features and classes, which can lead to complex decision boundaries, we examined six non-linear models. Previous studies have shown the efficacy of these non-linear models in microbiome-based host phenotype predictions.^4,5^ The non-linear models evaluated were decision tree (DT), k-nearest neighbors (kNN), artificial neural network (ANN), radial basis function kernel support vector machine (Radial SVM), random forest (RF), and extreme gradient boosting classifier (XGB). Notably, RF and XGB are ensemble learning models, built from multiple weak predictors, known for their robust performance.

**Figure 5.**
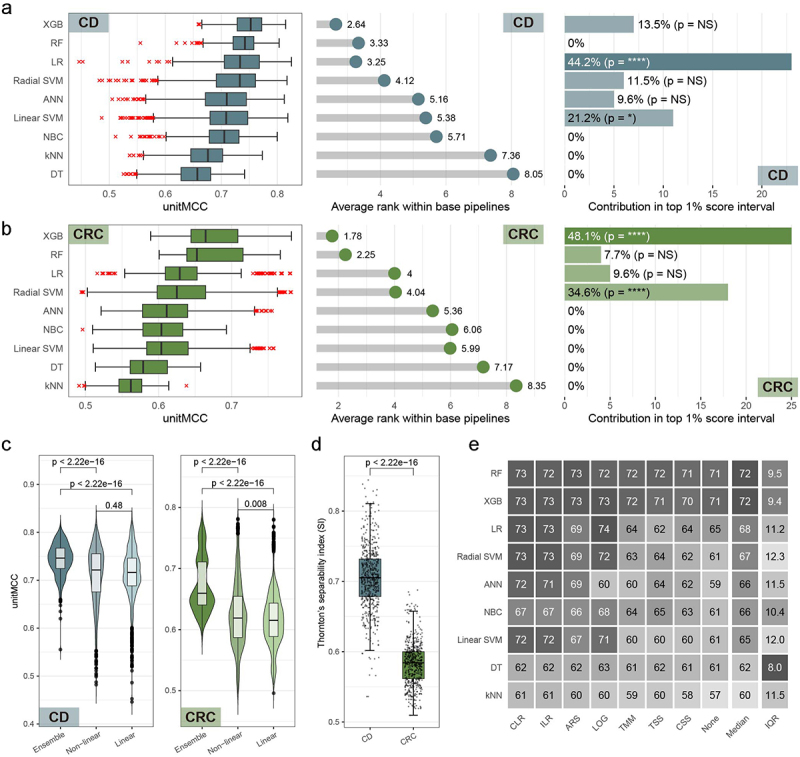
Assessment of classification models. (a-b) for both Crohn’s disease (CD) and colorectal cancer (CRC) diagnosis, distribution of unitMCC scores across 576 base pipelines and average rank within 576 base pipelines were plotted for each classification model (left and central panel). Additionally, bar plots illustrate contribution of each classification model to the top 1% best-performing ML pipelines (right panel) for CD (a) and CRC (b). These models are ordered in the plots by their descending median unitMCC values. Enrichment significance in top pipelines was determined with Fisher’s exact test, where ‘NS’ denotes *p* > 0.05; ‘*’ for *p* < 0.05; ‘**’ for *p* < 0.01; ‘***’ for *p* < 0.001; and ‘****’ for *p* < 0.0001. (c) The unitMCC distribution for ML pipelines in diagnosing CD and CRC is depicted in violin plots. We grouped the pipelines into three categories based on model architecture: ensemble models (‘Ensemble’ – RF and XGB), non-linear models (‘Non-linear’ – Radial SVM and ANN), and linear models (‘Linear’ – LR, Linear SVM, and NBC). Statistical significance across these groups was assessed using the Mann-Whitney U test. (d) Box plots show the geometric separability index of the training dataset after batch correction and normalization for all 5,184 ML pipelines. The Mann-Whitney U test was used to determine statistical significance. (e) We aggregated pipeline performance data for CD and CRC, categorizing it by ML model architecture and normalization methods. For clarity, unitMCC values were scaled by a factor of 100. Different shades indicate performance levels, with darker colors representing lower values in interquartile ranges (IQR). Both rows and columns are arranged in descending order of median performance.

In terms of overall performance, XGB surpassed all other classifiers in diagnosing both diseases (Figure 5a-b, left and central panels). The RF, another ensemble model, ranked second but was significantly less effective than XGB (*p* = 1.335e–07 for CD and *p* = 0.01638 for CRC). This aligns with previous findings in WMS-based prediction of host characteristics.^5^ Furthermore, we analyzed the effectiveness of WMS-based disease diagnosis across different decision boundary types of the ML models (Figure 5c). For the analysis of non-linear classifiers, we categorized ensemble models separately and excluded kNN and DT due to their lower performance, to prevent skewing the results. We observed that non-linear ensemble models significantly outperformed other types in diagnosing both diseases. Meanwhile, non-linear models without ensemble techniques also exceeded linear models in disease diagnosis, though the advantage was marginal for CD.

Contrasting with their impact on overall performance, the contribution of each classification algorithm to the top-performing ML pipelines varied between the two diseases. For CRC diagnosis, non-linear models, specifically XGB and Radial SVM, were predominant in the top 1% ML pipelines (Figure 5b, right panel). Conversely, for CD, linear models like LR and Linear SVM were more frequently contribute to the top 1% pipelines (Figure 5a, right panel). This trend aligns with our observation of only marginal advantages of non-linear models over linear models in CD prediction (Figure 5c), suggesting disease-specific preferences in effective ML model types.

The variation in effectiveness of WMS-based disease diagnosis between CD and CRC might stem from the differing properties of their gut microbiome profiles. Specifically, CD profiles could be more linearly separable than those of CRC. To explore this hypothesis, we evaluated the separability of input microbiome profiles across all composite pipelines using Thornton’s Separability Index (SI)^24^ (Figure 5d). SI measures how well two classes are distinguished in the feature space, with higher values indicating clearer separation. In line with the observed classification performance disparities, the median SI for CD datasets was notably higher than that for CRC datasets. This implies that microbiome profiles of CD patients are more distinctly distributed, hence more easily distinguishable. Consequently, a simple linear classifier like LR managed to achieve top classification results in CD diagnosis, reflecting the inherent linear separability within CD microbiome profiles.

Next, we broadened the analysis of performance distribution across different normalization methods to evaluate the combined effects of model architecture and feature engineering techniques (Figure 5e). Considering the combined performance across CD and CRC pipelines, we recommend CLR as the optimal data normalization method for RF, XGB, Radial SVM, ANN, Linear SVM, and kNN. For LR, NBC, and DT, the LOG is advised as the best practice for data normalization. When comparing the overall performance for both CD and CRC, we found that the generally top-performing classification models, RF and XGB, maintained high performance across all normalization methods. This can be attributed to their scale-invariant architectures and ability to automatically select important features during model construction, making them well-suited for high-dimensional datasets.

Other classification models like LR, Radial SVM, ANN, and Linear SVM, however, showed limited effectiveness when paired with sequencing depth-normalized datasets (TMM, TSS, CSS) or unnormalized data. While these models can handle high dimensions and have built-in regularization to prevent overfitting, their sensitivity to feature scale resulted in suboptimal overall performance. ANN showed reduced effectiveness with LOG normalization, possibly due to information loss or incompatibility in the normalization process. NBC, lacking a feature discrimination mechanism but insensitive to feature magnitude, performed consistently across various normalization methods, though it could not surpass the performance of other models. DT and kNN, the least effective models, are prone to the curse of dimensionality. Despite DT’s scale invariance and regularization, it can be overfit in high-dimensional datasets with complex decision boundaries. kNN struggles in high-dimensional, sparse spaces where distances between data points become less meaningful.^25^

In summary, while non-linear models, particularly ensemble models, generally excel in WMS-based disease diagnosis, they may not always yield the best ML pipelines when combined with various data types and preprocessing methods. Their scale invariance makes them practical choices for datasets with diverse feature scales. However, for diseases with linearly separable microbiome profiles, linear models with appropriate regularization techniques should also be considered to achieve peak performance. To offer guidance on the best practices for diagnosing diseases based on gut microbiome data, we recommend specific data processing pipelines for each machine learning algorithm (Supplementary table 2 and Supplementary figure 1–2).

### Optimal ML pipelines for disease diagnosis effectively generalize to unseen WMS data

We have rigorously evaluated different methods for each element of ML pipelines tailored for WMS-based diagnosis of gastrointestinal diseases like CD and CRC. Through systematic LODOCV, we identified the most effective methods for each pipeline element in diagnosing CD and CRC. Based on this, we hypothesized that a composite ML pipeline comprising these top-performing methods would excel in diagnosing diseases with unseen WMS data.

To test this, we constructed the optimal ML pipeline for each disease, selecting methods that contributed most to the top 1% ML pipelines. For both diseases, we chose taxonomic features based on gut-specific reference genomes (Tax-S+R) for profiling. Batch effects were uncorrected for CD and adjusted with ComBat-seq for CRC. The feature matrices underwent compositional normalization (ILR for CD, CLR for CRC). Finally, LR, a linear classifier, was selected for CD, and XGB, a non-linear ensemble classifier, for CRC.

**Figure 6.**
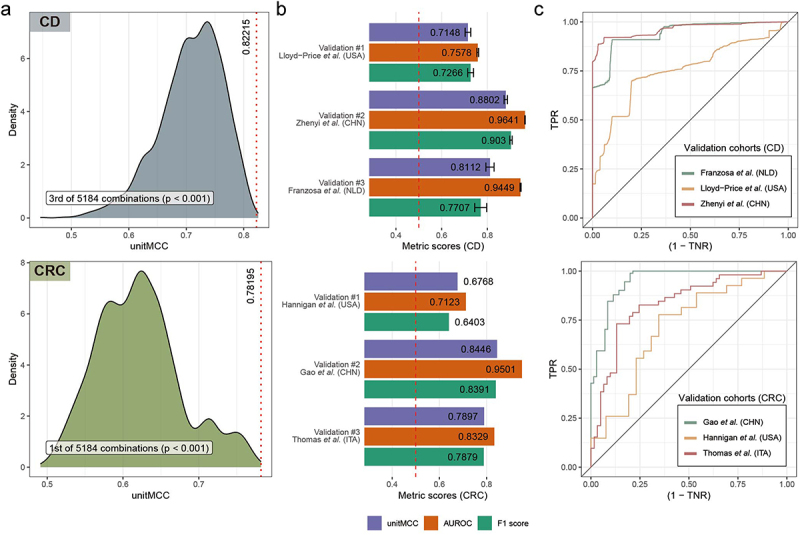
Performance analysis of the optimal ML pipelines and their validation. (a) Kernel density plots were used to illustrate the performance distribution of ML pipelines for Crohn’s disease (CD) (upper panel) and colorectal cancer (CRC) (lower panel) diagnoses. The optimal composite pipeline’s performance is highlighted with red dashed lines. The performance rank of the optimal pipelines and their statistical significance, as determined by the one-sample signed test, are noted at the bottom of each panel. (b) We evaluated binary classification metrics using three separate holdout validation datasets for CD (upper panel) and CRC (lower panel). Metrics averaged across 100 model iterations are displayed to the right of each bar. Red dashed lines represent the expected performance of a random classifier (0.5) in terms of unitMCC and AUROC. Notably, error bars are absent in the lower panel due to the uniform outcomes from all iterations, reflecting the deterministic nature of the XGB under the current parameter settings. (c) The Receiver Operating Characteristic (ROC) curve for the classification of the three holdout validation datasets is shown for both CD (upper panel) and CRC (lower panel). The average ROC curve from 100 model iterations is presented, with the black solid line indicating the baseline performance of a random classifier. Notably, the averaging process did not smooth the ROC curve shape in the lower panel, as mentioned in (b), due to the consistent performance of the XGB.

We first assessed the optimal ML pipelines against all others to validate our method selection approach (Figure 6a). The optimal ML pipelines showed top-tier performance (ranking 3rd and 1st out of 5,184 pipelines for CD and CRC, respectively, *p* < 0.001). Then, we evaluated these pipelines on new, unseen WMS datasets using three holdout validation cohorts for each disease (Figure 6b-c). The models were trained on the entire sample set of discovery cohorts 100 times to account for initialization randomness, and their performance was measured on each validation cohort using averaged unitMCC, F1 score, ROC curve, and AUROC values. Optimal pipelines for both diseases accurately classified microbiomes from diverse geographical origins. For CD, our top ML pipeline, which achieved a unitMCC of 0.82, demonstrated performance metrics of 0.72, 0.88, and 0.81 for validation sets #1, #2, and #3, respectively (Figure 6, upper panels). This indicates a variance of less than 12% from the optimal ML pipeline performance (−0.1, +0.06, −0.01, respectively), yet all instances exhibited solid diagnostic capabilities with acceptable levels of variance. A similar pattern was observed for CRC (Figure 6, lower panels) with a variance of less than 13% from the best ML pipeline (−0.1, +0.06, +0.01). These findings affirm that selecting best-performing methods for each element of ML pipeline leads to highly robust and generalizable models for WMS-based disease diagnosis.

Additionally, we explored the versatility of ML models by assessing whether those developed for CD could diagnose CRC, and vice versa. The optimal ML pipelines for both diseases were tested across diverse validation datasets. The results showed that while each pipeline performed best with its respective disease dataset, they also achieved reasonable accuracy in cross-disease predictions between CD and CRC (Supplementary figure 3). This suggests that despite being customized for specific diseases, the ML pipelines have the potential to predict other gastrointestinal disorders with a commendable level of precision.

### Optimal ML pipelines for WMS-based disease diagnosis uncover novel bacterial species associated with diseases

Typically, disease-associated microbes are identified by statistically analyzing their differential abundance between patients and healthy controls. These microbes, showing varied abundance, can serve as metagenomic biomarkers for diseases. While they may be either protective or pathogenic, it is important to note that not all disease-associated microbes exhibit changes in abundance between these two groups.

Some classification models reveal key features that are crucial for understanding the interplay between the microbiome and host phenotype. To explore whether important features of the diagnostic ML models could enhance the identification of disease-associated taxa beyond traditional statistical methods, we used Shapley Additive Explanations (SHAP).^26^ This model-agnostic approach estimates the contribution of each taxonomic feature to the model, offering an intuitive understanding of feature importance. SHAP values are particularly valuable as they adhere to properties like efficiency and additivity. We calculated SHAP values for bacterial species using the optimal ML pipelines for diagnosing CD and CRC (Supplementary table 3). Since the ILR-normalized profiles, the best normalization method for CD, lose direct correspondence with original profiles, we used CLR normalization for feature importance analysis. This alternative approach demonstrated comparable performance to the optimal pipeline, both in terms of relative performance among all composite pipelines and in validation with unseen data (Supplementary figure 4).

**Figure 7.**
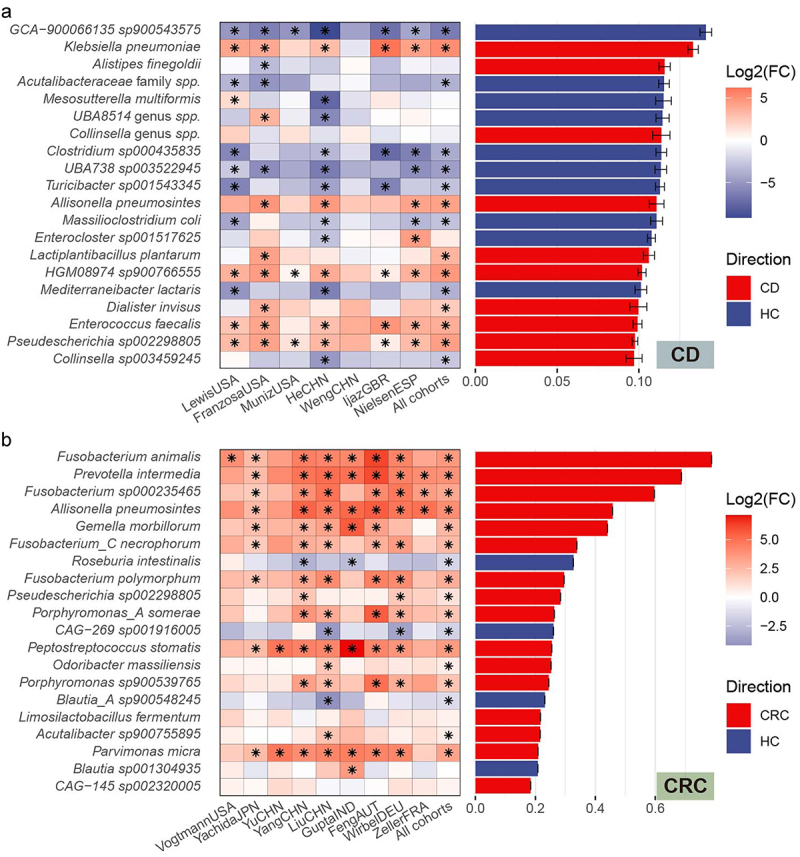
Investigating key species in diagnostic models. (a-b) the analysis is presented in two parts for each disease model – Crohn’s disease (CD) and colorectal cancer (CRC): Left Panel: We created heatmaps to showcase the fold changes in the top 20 important species across discovery cohorts and the batch-corrected, integrated cohort. These heatmaps illustrate the relative abundance changes of these species between patients and healthy controls. Right Panel: This section features bar plots of the absolute average Shapley additive explanations (SHAP) values for these top species. These values were computed from 100 independent model instances and then averaged. An asterisk denotes statistical significance in differential abundance (adjusted *p*-value <0.01 according to the Benjamini-Hochberg procedure). (a) For the CD diagnostic model, the left panel displays the differential abundance of the top 20 species, while the right panel shows their corresponding feature importance. (b) Similarly, for the CRC diagnostic model, differential abundance and feature importance of the top 20 species are shown. In both models, the absence of error bars in the lower panel is due to uniform outcomes across all iterations, a result of the XGB’s deterministic behavior with the current parameter settings.

In our analysis for each disease, we focused on the top 20 important species and their differential abundance between patients and healthy controls across discovery cohorts, utilizing the MaAsLin2 tool.^27^ Our findings revealed that in both diseases, these top 20 species exhibited significant differences in abundance in many cohorts (Figure 7a-b). Interestingly, we observed that even the most important species for diagnosis in both diseases only showed changes in abundance between patients and controls in certain cohorts. Particularly notable in the CD diagnostic model were species like *Mesosutterella multiformis*, *UBA8514 genus spp*, and *Enterocloster sp001517625*, which displayed differential abundance in opposite directions across different cohorts. For instance, *M. multiformis* significantly increased in abundance in patients from a USA cohort, while it decreased in patients from a China cohort (see Figure 7a). These findings highlight the strength of ML approaches when applied to multi-cohort datasets, as they can identify more robust and generalizable disease-associated species that may not be apparent through traditional single-cohort statistical analyses.

Interestingly, our analysis revealed a distinct pattern in the top 20 disease-associated species for each diagnostic model. In the CD diagnostic model, many of the key species were identified as protective, while in the CRC diagnostic model, most of the crucial species were pathogenic. This suggests that the gut microbiome in CD patients is more influenced by the abundance of protective species, whereas in CRC patients, the abundance of pathogenic species plays a more significant role. This distinction has important implications for treatment strategies. For CD, it appears that restoring a healthy microbiome could be a more effective approach, emphasizing the need to support and enhance the presence of protective species. Conversely, for CRC treatment, strategies focused on protecting against pathogenic microbes, or pathobionts, might be more beneficial.

In our subsequent analysis, we explored whether the key species identified by the ML models could uncover novel disease-associated bacterial species. For CD, the second most important species, *Klebsiella pneumoniae*, is already known to be linked to CD development.^28,29^ However, most of the other top 20 species have not been previously recognized as associated with CD, suggesting new potential disease-related gut bacterial species.

Conversely, in the case of CRC, the majority of the top 20 important species are already well-established as disease-associated. Notably, species from the *Fusobacterium* genus, such as *F. animalis*, *F. sp000235465*, *F._C necrophorum*, *F. polymorphum*, were among the highest-ranked. *F. animalis* and *F. polymorphum* are subspecies of *F. nucleatum*, a key bacterium associated with CRC.^30,31^ The involvement of *Fusobacterium* species, particularly *F. nucleatum*, in the initiation, development, and metastasis of CRC is well-documented, with a noted abundance in tumor tissues.^32,33^ Other recognized CRC-associated species like *Prevotella intermedia*,^34^ *Allisonella pneumosintes* (also classified as *Dialister pneumosintes* in the NCBI taxonomic hierarchy),^35,36^ and *Gemella morbillorum*^37,38^ were also highly influential in the model. Among the four protective species for CRC, only *Roseburia intestinalis* was previously reported to have a negative association with CRC,^39,40^ whereas *CAG-269 sp001916005*, *Blautia_A sp900548245*, and *Blautia sp001304935* lacked significant prior research. These findings suggest that in CRC, the proliferation or invasion of certain key species may play a critical role in cancer development and progression, contrasting with the pattern observed in CD where less-known species emerged as significant.

## Discussion

In the present study, we thoroughly assessed how different aspects, including the microbiome profile modality, batch correction method, normalization approach, and choice of classification algorithm, influence the effectiveness of ML pipelines for disease diagnosis using WMS data. We then constructed an optimal ML pipeline by integrating the best-performing methods and further validated its generalizability on unseen data from various geographical cohorts. Drawing from the insights of this study, we provide several key guidelines for developing effective ML models tailored to WMS-based disease diagnosis.

Firstly, we recommend prioritizing gut-specific, species-level taxonomic features when constructing ML models for disease diagnosis using fecal sample WMS. Recent research has suggested that functional features might offer more generalizability across individuals,^41–43^ and potentially superior performance in disease classification compared to taxonomic features.^44^ However, our findings indicate a clear superiority of taxonomic features over functional features in this context. Furthermore, another recent study exploring ‘multi-view learning’, which integrates both taxonomic and functional features, found only marginal differences in performance compared to classifiers using solely taxonomic profiles. This suggests a potential redundancy in the information gleaned from taxonomic and functional features derived from the same metagenomic sequences.^45^ Therefore, for feces sample WMS-based disease diagnosis, focusing on species-level taxonomic features may offer a more effective approach for ML model construction.

Secondly, when mitigating unwanted batch effects in your data, it is crucial to avoid overcorrecting to the extent that disease-relevant differences are lost. Our benchmarking analysis revealed that the effectiveness of batch correction tools largely hinges on their ability to preserve variance related to the disease during the adjustment process. Thus, we recommend employing a combination of validation metrics, such as LISI or the PERMANOVA test. These tools can help identify a batch correction method that minimizes batch effects while maintaining crucial differences between groups in your dataset.

Thirdly, in line with previous findings in 16S microbiome studies,^4^ we found that compositional transformations, specifically ILR and CLR, were most effective in addressing the inherent challenges of microbiome data. Both methods performed similarly well and transformed the data into a real vector space, suitable for analysis using standard multivariate methods. However, each transformation has its limitations: CLR transformation can lead to a singular covariance matrix, creating collinearity among the adjusted features. In contrast, ILR transformation reduces the data dimensionality by one, complicating the interpretation as it disrupts the one-to-one correspondence between pre- and post-transformation features. Considering these characteristics, we recommend employing CLR for feature importance analysis post-ML model training, as it facilitates easier interpretation of individual features. On the other hand, ILR is preferable for ordination analyses that require beta diversity calculations, such as Principal Coordinate Analysis (PCoA), where preserving the relative differences between samples is crucial.

Fourthly, to achieve optimal disease classification in diagnostic ML models handling sparse, over-dispersed, and high-dimensional microbiome data, two key features have emerged as particularly important: the built-in mechanism for prioritizing important features, and scale invariance. Classification models like RF and XGB excel in these areas. They automatically select significant features through processes such as impurity reduction or gradient descent optimization, which is crucial for handling high-dimensional data filled with potentially irrelevant features. Additionally, their scale invariance, owing to the model construction from multiple decision trees that compare features against specific thresholds, allows them to remain unaffected by the scale of the input features. This makes them perform well across various normalization methods. Consequently, we advocate for the use of ensemble models like RF and XGB in most scenarios for their optimal classification performance and minimal requirement for feature preprocessing. Linear models with proper regularization techniques can also be considered for efficiency and simplicity, especially after assessing the linear separability of the data.

While our benchmark study offers valuable insights and empirical guidelines for fecal sample WMS-based disease diagnosis, it is important to consider several limitations. Firstly, the study did not incorporate comprehensive clinical and demographic data, such as disease state, severity, age, sex, and body mass index, due to limitations in the available metadata from public databases. This omission is significant given the profound influence of genetic, environmental, and clinical factors on gut microbiota composition.^46^ Future research would benefit from including these variables for a more robust model. Secondly, our stringent criteria for selecting metagenomic samples, including the exclusion of antibiotic-treated samples and focusing only on baseline fecal metagenomes, likely reduced technical biases, potentially affecting the perceived efficacy of batch correction tools in preserving disease-related variance. Thirdly, the hyperparameters of ML models were not optimized; they were set to a basic level without prior dataset knowledge, assessing only fundamental performance of the models. Real-world applications, however, would necessitate fine-tuning these hyperparameters and implementing appropriate cross-validation procedures to enhance model performance. Lastly, the study cannot conclusively determine whether the identified biomarkers through feature importance analysis are causative or consequential to the diseases. Many of these biomarkers are also poorly characterized due to a lack of research or culturing challenges, underscoring the need for their validation through further in vitro and in vivo studies and a more comprehensive understanding of the gut microbiome landscape. These limitations underscore the importance of a nuanced and comprehensive approach in future research to refine WMS-based disease diagnosis models.

## Materials and methods

### Collection of metagenomic datasets from the public databases

Raw sequencing reads of 10 and 12 case-control datasets for CD and CRC, respectively, obtained from 21 published studies were downloaded from the NCBI Sequence Read Archive (NCBI SRA).^47^ Metadata, including disease status, age, sex, and body mass index of each sample, were acquired from the corresponding publication’s supplementary materials and the curatedMetagenomicData v3.6.2.^48^ To minimize non-biological biases arising from demographical variability and technical artifacts, only high-quality metagenomic samples meeting the following criteria were included in this benchmark: (i) Whole metagenome sequencing reads of the human fecal samples. (ii) Cross-sectional or baseline samples (in case of longitudinal studies). (iii) Samples without any antibiotic treatment within three months before stool collection, if clinical metadata is available. For CRC datasets, samples annotated as ‘adenoma’, or ‘advanced adenoma’ were excluded from the analysis. Clinical subtypes of CRC were not considered due to lack of availability of clinical metadata. Samples annotated with two or more diseases were also excluded.

### Preprocessing of raw sequencing reads

Raw metagenomic reads were first trimmed to remove any low-quality base pairs and remaining adapter sequences using BBDuk v38.58 with the following parameters: ktrim = r hdist = 1, k = 19, mink = 11, qtrim = rl, trimq = 10, ml = 36 tbo tpe. The trimmed reads were then aligned to the GRCh38.p13 human reference genome to remove any human-originated contaminant reads using Bowtie2 v2.5.1^49^ and Samtools v1.13.^50^ Following the preprocessing steps, metagenomic samples with insufficient read depth (less than 1 million reads after preprocessing)^51^ or highly contaminated (host contamination rate ≥ 50%)^52^ were excluded from downstream analyses.

### Taxonomic and functional feature profile modalities

Three different methods for taxonomic classification of sequence reads, along with three distinct microbial reference databases, were employed in this study. Consequently, five unique modalities for taxonomic profiling were created and assessed, each based on a different combination of these read classification methods and reference databases (Table 1). For more in-depth information about taxonomic profiling methods, please refer to the Supplementary Methods section.

Functional profiling was based on either pathway or gene family. MetaCyc^53^ pathway abundances were measured from quality-controlled reads using HUMAnN v3.6^54^ with default parameters. Gene family abundances were measured with either HUMAnN along with the latest compatible pangenome and protein databases (mpa_vJan21_CHOCOPhlAnSGB_202103 for nucleotide alignment; uniref90_201901b_full for translated alignment) or HRGM gene family. Detailed descriptions of the functional profiling methods can be found in the Supplementary Methods section.

### Leave-one-dataset-out cross-validation (LODOCV)

To evaluate ML pipeline performance with a focus on cross-cohort generalizability, the LODOCV was used. Briefly, for each study in the full feature matrix, a CV fold feature matrix was compiled using that study as the test set and the remaining studies as the training set. The features of each CV fold matrix were then filtered without prior knowledge, retaining only features that have non-zero values in more than 10% of the sample in more than one study among the training set, to reduce sparsity. Afterward, batch correction, normalization, and model training procedures were conducted separately using each filtered CV fold matrix. To simulate a real-world diagnostic situation, where new unlabeled samples collected from patients and classified using a model trained on preexisting datasets, batch correction and normalization procedures were applied to all samples included in the CV fold matrix by providing only batch information, without biological covariates. Classification models were constructed and evaluated using the corrected training and test set samples.

### Correcting for batch effects across cohorts

The order of batch correction and normalization was set differently for each combination of methods to account for the different assumptions of the underlying distribution and required format of input values for each batch correction method. We employed the following methods for batch correction: naïve batch mean centering, ComBat-seq,^11^ LIMMA,^12^ MMUPHin,^13^ ConQuR.^14^ More in-depth information about each method is available in the Supplementary Methods section.

### Additional analyses of batch correction methods

Explained variance (R^2^ values) by disease and batch label of the batch-corrected dataset with each batch correction method were calculated using PERMANOVA test implemented as adonis2 function from the vegan v2.6–4 with the following modification to default parameters: method=‘robust.aitchison’. To calculate LISI values of the batch-corrected dataset with each batch correction method, pairwise robust Aitchison distances were first calculated with vegdist function from the vegan v2.6–4 with the following modification to default parameters: method=‘robust.aitchison’. iLISI and cLISI values were calculated using compute_lisi function from the lisi v1.0^17^ with default parameters and subsequently normalized to fall in 0–1 range.

### Data normalization and transformation

We employed the following methods for data normalization and transformation: Total sum scaling (TSS), cumulative sum scaling (CSS),^20^ trimmed mean of M-values (TMM),^21^ centered log-ratio transformation (CLR),^22^ isometric log-ratio transformation (ILR),^23^ arcsine square root transformation (ARS), log transformation, handling zero values for log-based normalization techniques. More in-depth information about each method is available in the Supplementary Methods section.

### Classification models for disease diagnosis

Classification models evaluated in this study were implemented using scikit-learn v1.2.0,^55^ except for the gradient boosting tree model implemented with xgboost v1.7.2.^56^ Hyperparameters were selected before model training without any optimization. We employed the following algorithms for binary classification: logistic regression, naïve Bayes classifier, support vector machine (SVM), k-nearest neighbors (kNN), decision tree, random forest, gradient boosting tree, artificial neural network. More in-depth information about each classification model is available in the Supplementary Methods section.

### Calculation of Thornton’s separability index

Thornton’s separability index (SI) was calculated using an in-house Python script for processed input datasets for all ML pipelines, as defined in the original paper:

$$\mathrm{SI}=\frac{\sum_{i=1}^{n}\left(f\left(x_{i}\right)+f\left({x{\;}^{\prime }}_{i}\right)+1\right)\mathrm{mod}2}{n}$$

where $x^{\prime }$ is the nearest neighbor of point x and n is the number of data points.

### Classification performance evaluation

The overall performance of each pipeline was assessed via unit-normalized Matthew’s correlation coefficient (unitMCC). The unitMCC was calculated using matthews_corrcoef function from scikit-learn v1.2.0 and then further normalized by adding 1 to a value and then dividing the resulting sum by 2. The unitMCC score of each ML pipeline was calculated by averaging scores of all CV folds, weighted proportionally to the test set size of each fold to avoid overestimating scores computed on a relatively small test set.

### Selecting the optimal ML pipeline

Among methods for each ML pipeline element, the one that most contribute to the top 1% best-performing ML pipelines was selected and combined into an optimal ML pipeline. The statistical significance of the score of the best pipeline in the score distribution was assessed using a 1-sample sign test, performed using signTest function from EnvStats v2.8.0 with default parameters.

### Feature importance analysis

Feature importance of the optimal ML pipelines was calculated using Shapley additive explanations (SHAP) framework. The SHAP values of the optimal ML pipelines for CD and CRC diagnoses were computed using LinearExplainer and TreeExplainer^57^ classes, respectively, from shap v0.41.0 with default parameters.

### Differential abundance analysis of features

Statistical tests for the differential abundance of each feature were performed using Maaslin2^27^ function from MaAsLin2 v1.12.0 R package. Disease labels were set as the only fixed effect, and the following modifications were made to default parameters: min_prevalence = 0.0, minabundance = −1e + 08, normalization=“NONE”, transform=“NONE”. The input feature matrix was subjected to the normalization and batch correction method from the best pipeline combination before conducting the tests.

## Supplementary Material

Supplementary figure 4.jpg

Supplementary figure 2.jpg

Supplementary table 3.xlsx

Supplementary figure 3.jpg

Lee_MBiomeML_Suppl materials_forGM_rev_20240416.docx

Supplementary figure 1.jpg

## Data Availability

The authors confirm that the data supporting the findings of this study are available within its supplementary materials (Supplementary table S1).
