# Supplementary figures and images for: Comprehensive assessment of machine learning methods for diagnosing gastrointestinal diseases through whole metagenome sequencing data
Source: Gut Microbes. 2024 Jul 7;16(1):2375679. doi: 10.1080/19490976.2024.2375679 (PMC11229738; doi:10.1080/19490976.2024.2375679)

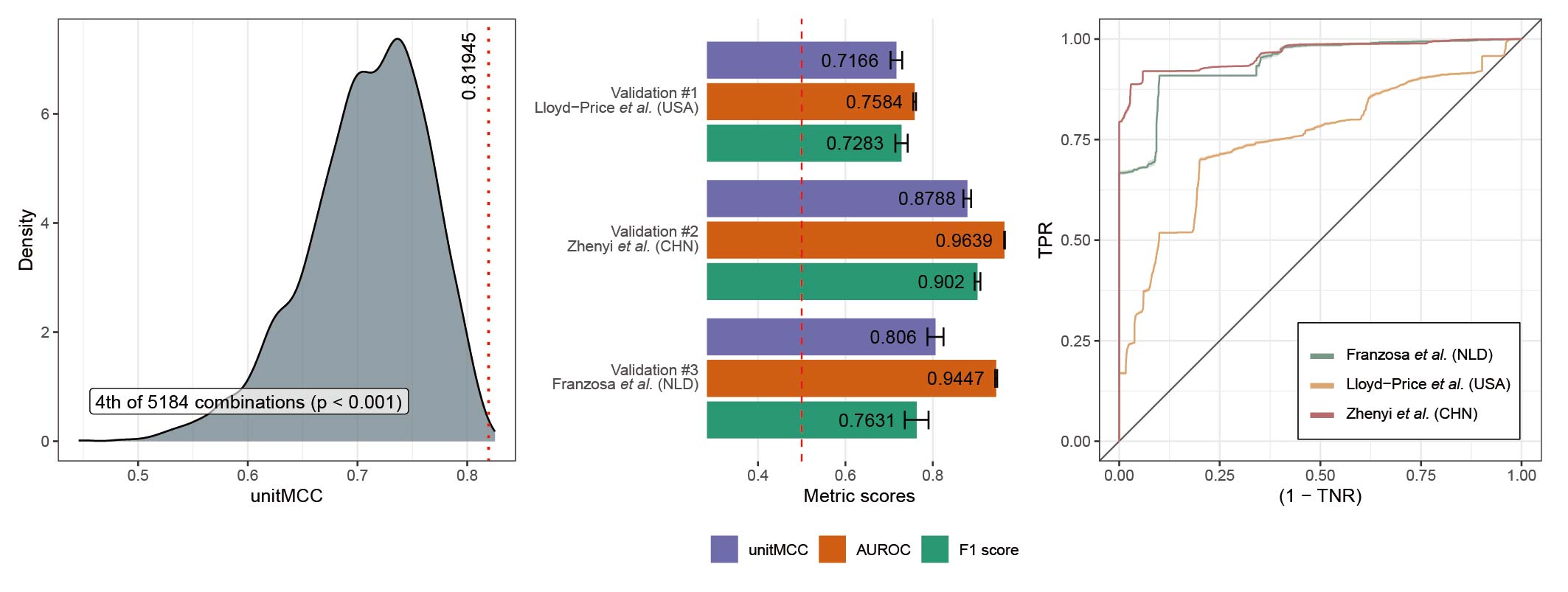

Supplement: Supplementary figure 4.jpg [file KGMI_A_2375679_SM2141.jpg]

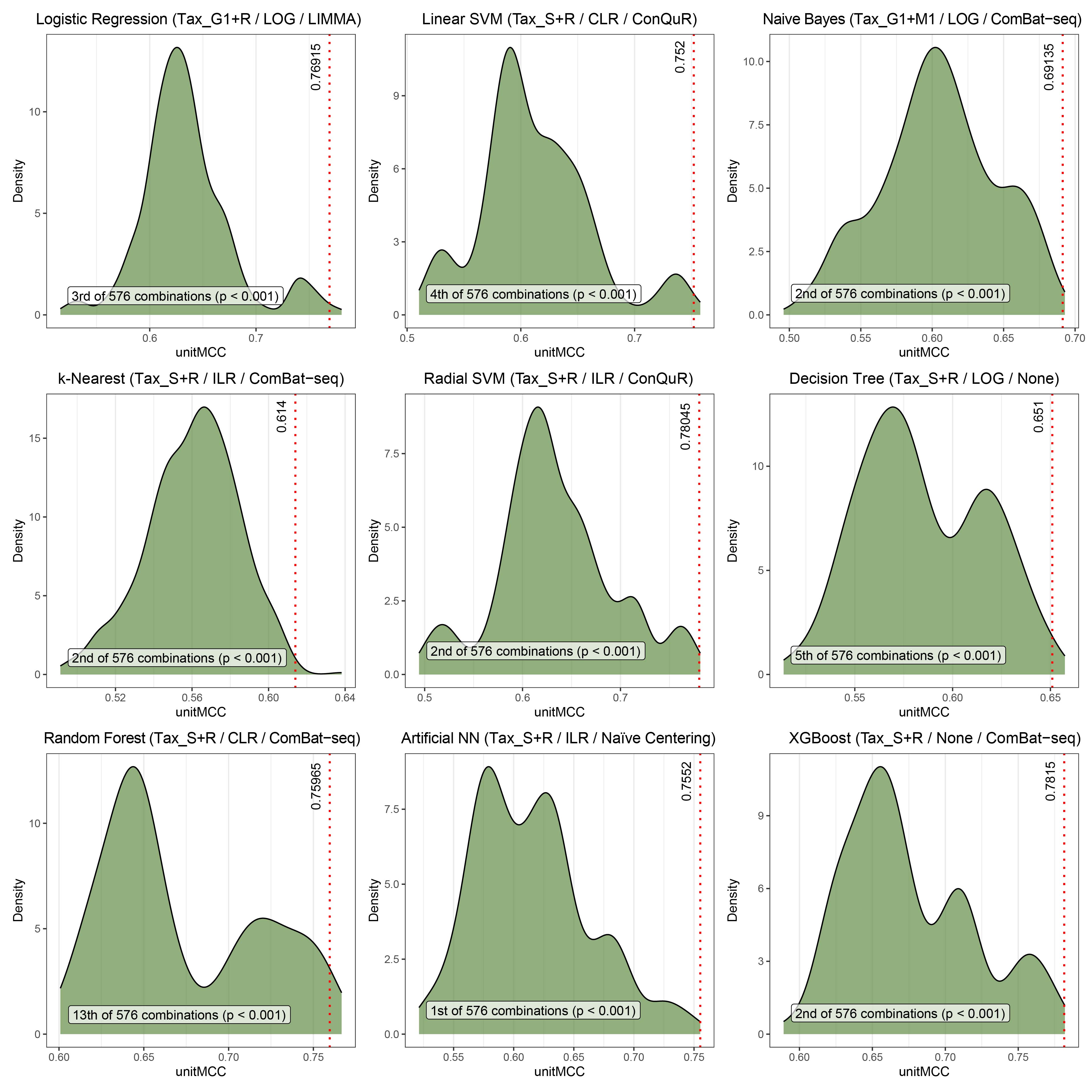

Supplement: Supplementary figure 2.jpg [file KGMI_A_2375679_SM2140.jpg]

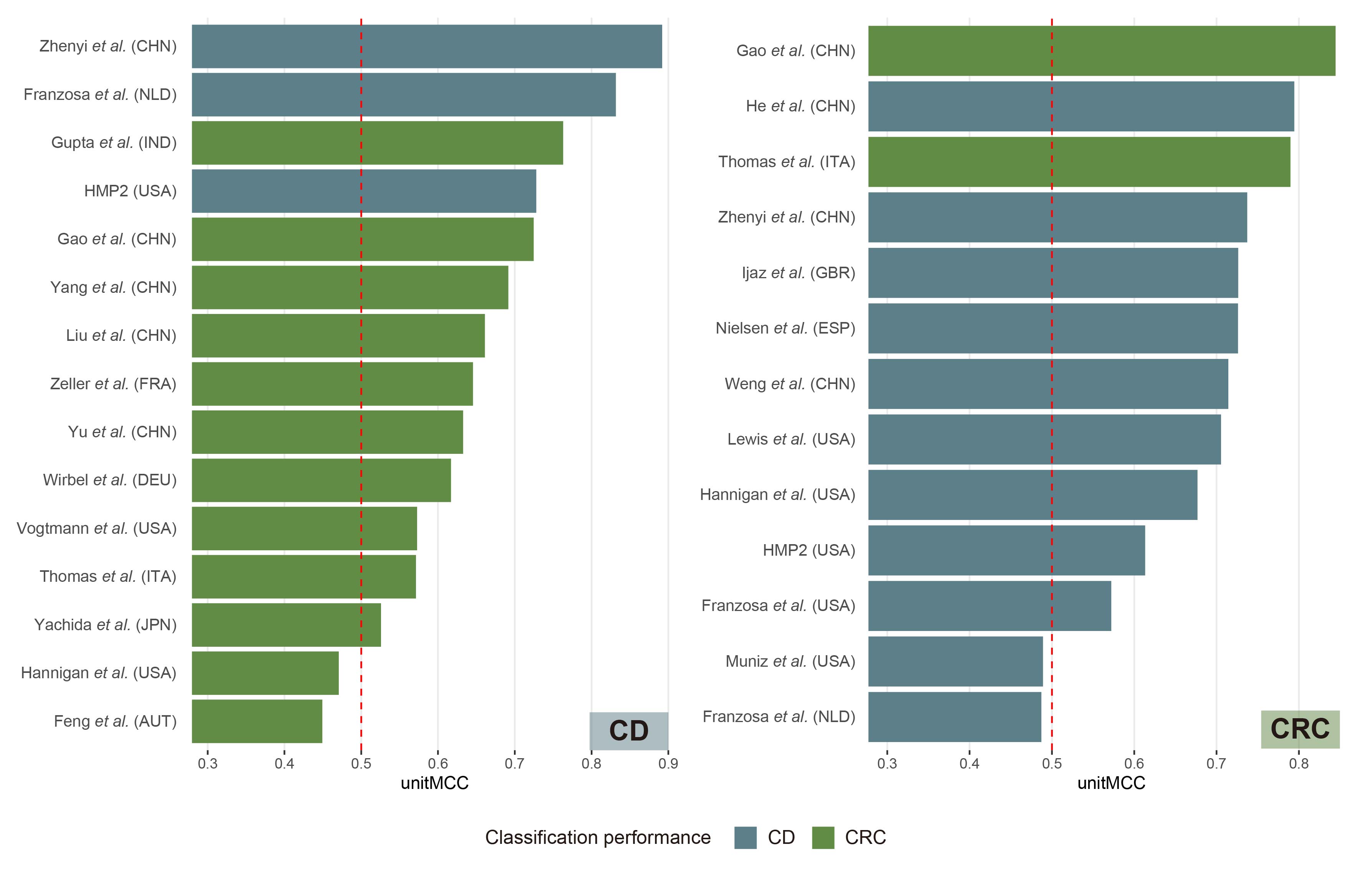

Supplement: Supplementary figure 3.jpg [file KGMI_A_2375679_SM2138.jpg]

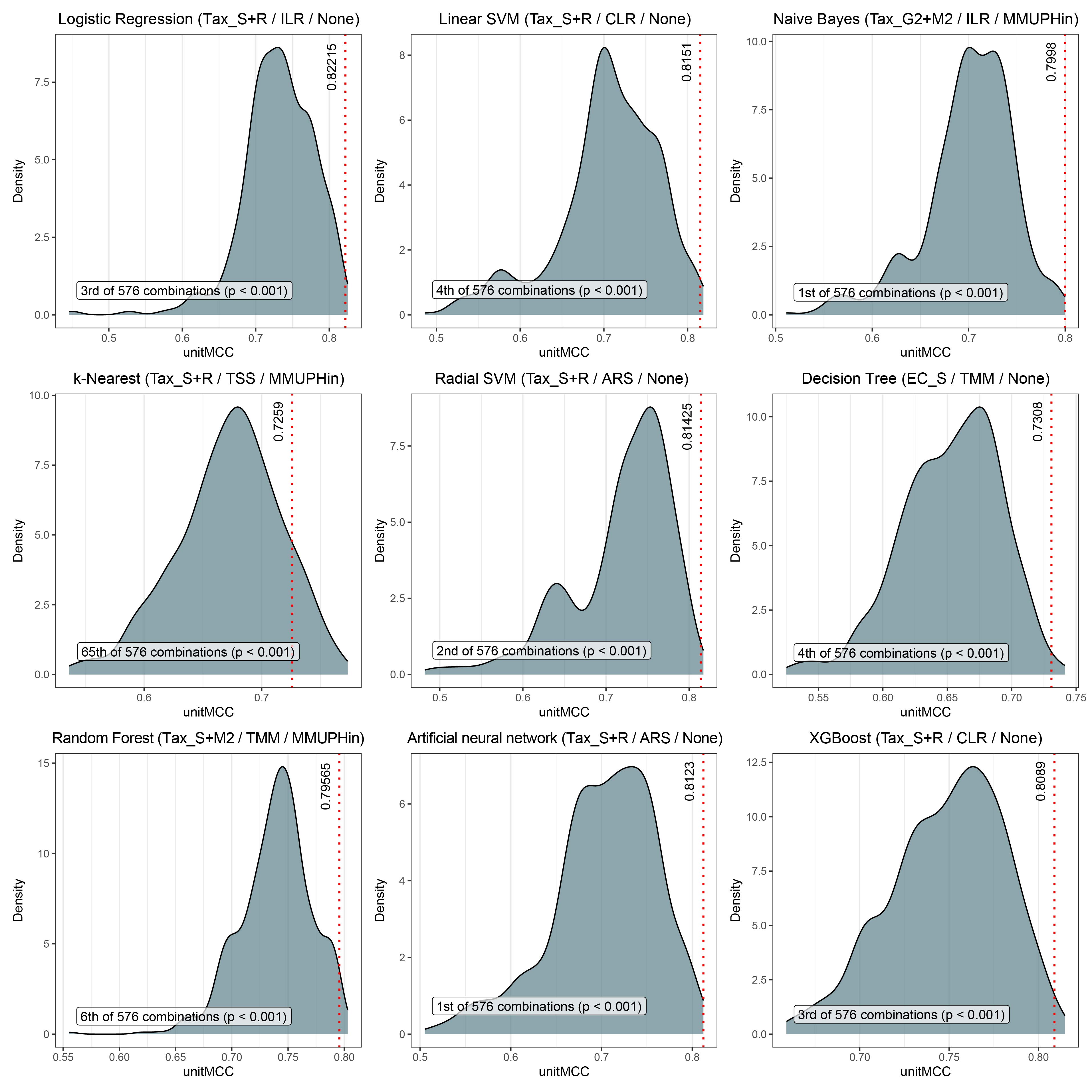

Supplement: Supplementary figure 1.jpg [file KGMI_A_2375679_SM2136.jpg]
