# Supplementary material for: Comprehensive assessment of machine learning methods for diagnosing gastrointestinal diseases through whole metagenome sequencing data
Source: Gut Microbes. 2024 Jul 7;16(1):2375679. doi: 10.1080/19490976.2024.2375679 (PMC11229738; doi:10.1080/19490976.2024.2375679)
Supplement: Lee_MBiomeML_Suppl materials_forGM_rev_20240416.docx [file KGMI_A_2375679_SM2137.docx]

***Supplementary Materials***

**Comprehensive assessment of machine learning methods for diagnosing gastrointestinal diseases through whole metagenome sequencing data**

Sungho Lee and Insuk Lee

**Contents**

- *Legend for supplementary figures*
- *Legend for supplementary tables*
- *Supplementary methods*
- *Supplementary references*

**Legend for supplementary figures**

**Supplementary figure 1-2.** Kernel density plots were used to illustrate the performance distribution of ML pipelines for Crohn’s disease (CD) (1) and colorectal cancer (CRC) (2) diagnoses using each machine learning algorithm. The suggested best practice pipeline’s performance is highlighted with red dashed lines. The performance rank of the optimal pipelines and their statistical significance, as determined by the one-sample signed test, are noted at the bottom of each panel.

**Supplementary figure 3.** The cross-disease diagnostic performance of the optimal ML pipelines for Crohn’s disease (CD) (right) and colorectal cancer (CRC) (left). While each pipeline achieves its best performance when diagnosing the disease it was trained on, it also demonstrates reasonably good performance in predicting other gastrointestinal disorders across various cohorts.

**Supplementary figure 4.** The relative performance of an alternative diagnostic ML model for Crohn’s disease (CD) to other ML pipelines and validation through previously unseen datasets. Figure layout and details are the same as for Figure 6.

**Legend for supplementary tables**

**Supplementary table 1.** Summary statistics of included studies and metagenomic samples in the benchmark.

**Supplementary table 2.** Suggested best data processing pipelines for each ML algorithm for each disease modeling.

**Supplementary table 3.** Full table of SHAP values for features included in diagnostic ML models Crohn’s disease (CD) and colorectal cancer (CRC).

**Supplementary methods**

**Microbiome profiling with taxonomic features**

***Methods for taxonomic classification of sequence reads***

**Kraken2 & Bracken** (R): Kraken2 ^1^ is a genome sequence-based taxonomic read classification system that utilizes exact k-mer matches to assign short genomic substrings to the lowest common ancestor (LCA) taxa. The software employs a compact hash table database containing key-value pairs of k-mers and their corresponding LCAs, enabling time and memory-efficient genome assignments. Compared to its predecessor, Kraken, the database build time, memory efficiency, and consequently, overall classification speeds, are greatly improved by further hashing k-mers into l-mer minimizers and introducing spaced seeds. Bracken ^2^ is a companion software of Kraken2 and estimates the actual number of reads originating from clades of a specific taxonomic level *e.g.*, species by re-distributing the classified reads using a Bayesian model.

**mOTUs3** (M1): mOTUs3 ^3^ is a phylogenetic marker gene (MG)-based taxonomic profiler that estimates the relative abundance of operational taxonomic units (mOTUs). mOTUs3 first aligns metagenomic reads to a pre-indexed database of MGs using the BWA-MEM ^4^ algorithm. The resulting alignments are then filtered based on nucleotide identity and alignment length, and abundances for each marker gene cluster (MGC) are calculated. Finally, the abundance of mOTUs is calculated as the median abundance of MGCs belonging to that mOTU.

**MetaPhlAn4** (M2): MetaPhlAn4 ^5^ is a MG-based taxonomic profiler designed for estimating the composition of host-associated and environmental microbial communities at the species level. MetaPhlAn4 maps metagenomic reads to the MG database using Bowtie2 and computes the coverage of detected taxonomic clades as the robust average of the coverage of clade-specific marker genes. The resulting taxonomic abundances are then normalized across the community.

***Databases of reference microbial genomes used for taxonomic profiling***

**HRGM v2** (S): It is an updated version of Human Reference Gut Microbiome (HRGM) ^6^ which is a catalog of human gut-specific reference microbial genomes. HRGM improves previous reference microbial catalogs, which are often biased towards culturable, abundant taxa and major geographic regions, by incorporating newly assembled genomes from previously underrepresented Asian populations and by utilizing ultra-deep sequenced (> 30Gbp or > 100 million read pairs) fecal metagenomic samples. The HRGM v2 (currently in manuscript preparation) further extends this advancement by introducing novel genome assemblies from yet underrepresented African populations and applying a more stringent genome quality filtering procedure. The database comprises 4,824 human gut-specific bacterial and archaeal species identified from 155,212 *de novo* assembled high-quality genomes (completeness > 90% and contamination < 5%).

**mOTUs-DB v3.0.3** (G1): The mOTUs database (mOTUs-DB) is a companion database of mOTUs3 profiler. It enables accurate MG-based taxonomic profiling of metagenomic samples, ranging from well-studied environments to various underexplored microbial niches. The latest release of the database comprises 33,570 mOTUs (a 2.36-fold increase compared to the previous major release, v2.5.1) of bacterial and archaeal origin, derived from 23 biological and ecological environments. This expansion was achieved by updating 499,512 *de novo* assembled genomes and external genomes, such as metagenome-assembled genomes (MAGs), isolated genomes, and single amplified genomes (SAGs) into the existing database. Three types of mOTUs compose the database: (1) 11,915 ref-mOTUs, which represent taxonomically annotated species, (2) 2,297 meta-mOTUs, representing unknown species obtained from metagenomic contigs, and (3) 19,358 ext-mOTUs, representing unknown species obtained from external, public source-derived MAGs.

**ChocoPhlAn vOct22** (G2): ChocoPhlAn is a clade-specific marker gene database tailor-made for MetaPhlAn profiler. The database enables comprehensive profiling of taxonomic abundances of microbial communities, including bacteria, archaea, and eukaryotes, from various host-associated or ecological environments. The database adopts species-level genome bins (SGBs) as primary taxonomic units, integrating curated reference genomes and MAGs of previously unseen, understudied taxa. This approach achieves both sensitivity of assembly-based approaches and the accuracy of reference-based methods. The state-of-the-art release, ChochPhlAn vOct22, incorporates over 5 million unique marker genes for 30,550 SGBs extracted from an extended compendium of ~ 1 million microbial genomes. The included SGBs were defined from either reference genomes of existing, well-studied species (known SGBs; kSGBs) or the MAGs of uncharacterized species (unknown SGBs; uSGBs).

***Five modalities for taxonomic profiling using whole metagenome sequencing reads***

**Tax_S+R**: Taxonomic profiling using a genome-based method, Kraken2 and Bracken (R), and a reference genome database, HRGM v2 (S), which is specific for human gut microbiota.

**Tax_G1+M1**: Taxonomic profiling using a marker-based method, mOTUs3 (M1), and a database of OUT-specific markers, mOTUs-DB v3.0.3 (G1), which encompass microbes from various environments (not specific for human gut).

**Tax_G2+M2**: Taxonomic profiling using a marker-based method, MetaPhlAn4 (M2), and a database of species-specific markers, ChocoPhlAn vOct22 (G2), which encompass microbes from various environments (not specific for human gut).

**Tax_S+M2**: Taxonomic profiling using a marker-based method, MetaPhlAn4 (M2), and a reference genome database, HRGM v2 (S), which is specific for human gut microbiota. For this pipeline, a custom database of species-specific markers was generated from HRGM v2 through the same process used for the ChocoPhlAn database^5^.

**Tax_G1+R**: Taxonomic profiling using a genome-based method, Kraken2 and Bracken (R), and a database of OUT-specific markers, mOTUs-DB v3.0.3 (G1). For this pipeline, all the genomes used for the mOTUs-DB construction were downloaded using the custom script provided from the authors.

We could not test Tax_G2+R, because the full genome sequences of the ChocoPhlAn were not publicly available.

***Taxonomic abundance profiling with Kraken2 and Bracken***

Quality-controlled reads were classified with Kraken2 v2.1.2 with the following modification to default parameters: --confidence 0.2. To maximize classification capabilities and consistency along the Genome Taxonomy Database (GTDB) ^7^ annotation structure, the database was constructed by incorporating all microbial genomes rather than reference genome sets, while assigning genomes annotated with the same GTDB species to strain-level nodes. Any other parameters for database construction were set to default. Following Kraken2 classification, species-level abundance in each sample was estimated with Bracken v2.8.0 using default parameters (-l S -t 0). The abundance estimates were then divided by the average genome length of the taxa to yield taxonomic read count.

***Taxonomic abundance profiling with MetaPhlAn***

Taxonomic abundances were estimated from quality-controlled reads using MetaPhlAn v4.0.6 with default parameters. The resulting relative abundance values of each species were multiplied by the number of reads of the sample to generate taxonomic read count-like abundance values before being merged into a single matrix.

***Taxonomic abundance profiling with mOTUs***

Taxonomic abundances were estimated from quality-controlled reads using mOTUs v3.0.3 with default parameters. The resulting relative abundance values of each species were multiplied by the number of reads of the sample to generate read count-like abundance values before merging into a single matrix.

**Microbiome profiling with functional features**

***Pathway abundance profiling with HUMAnN***

MetaCyc ^8^ pathway abundances were estimated from quality-controlled reads using HUMAnN v3.6 ^9^ with default parameters and the latest compatible pangenome and protein databases (mpa_vJan21_CHOCOPhlAnSGB_202103 for nucleotide alignment; uniref90_201901b_full for translated alignment). The resulting pathway abundance profiles were then merged into a single matrix by an in-house script without any modification.

***Gene family abundance profiling with HUMAnN***

The gene abundance table, a primary output of HUMAnN, was subjected to the utility script humann_regroup_table to generate gene family abundance for Gene Ontology (GO) ^10, 11^, Enzyme Commission number (EC) ^12^, and Kyoto Encyclopedia of Genes and Genomes (KEGG) Orthology (KO) ^13^ terms. The script was invoked with default parameters, except for the grouping options: ‘-g uniref90_go’ for calculating GO abundance, ‘-g uniref90_level4ec’ for EC abundance, and ‘-g uniref90_ko’ for KO abundance. The resulting gene family abundance profiles were then merged into a single matrix without any modification. For the GO abundance matrix, the merged matrix was additionally filtered to reduce hierarchical redundancy by removing any GO terms that have one or more child terms in the matrix, according to the latest GO annotation (go-basic ontology, release 2022-12-04).

***Gene family abundance profiling with HRGM***

For estimating HRGM gene family abundance, gene sequences of all conspecific genomes for each HRGM species-level taxon were first clustered into a pangenome using Panaroo v1.3.0 ^14^, a graph-based pangenome clustering tool, with the following parameters: --clean-mode strict -c 0.90 -f 0.5 --merge_paralogs --core_threshold 0.90. Resulting representative pangenome gene sequences were further clustered with Linclust algorithm implemented in MMseqs v13.45111 ^15^ to reduce redundancy. The clustered 90% identity pangenome sequences were functionally annotated with eggNOG-mapper v2.1.9 ^16^. Quality-controlled reads were then aligned using bowtie2 v2.5.1 aligner to the HRGM pangenome 90% identity protein database with ‘--very-sensitive’ mode and post-processed to handle sequence length variation, alignment quality, and multiple mapped reads. In both steps, alignment parameters and post-processing algorithm of HUMAnN software was adopted for consistency. For post-processing, aligned reads were converted into gene family abundance through the following steps:

1. Only high-quality alignments with a percent identity of more than 80% and query (read) coverage of more than 90% were considered valid and retained for abundance calculation.
2. The coverage of all aligned pangenome proteins were calculated. Proteins that are not covered above 50% of the sequence by valid alignments were discarded before the following abundance calculation.
3. For each valid alignment to a pangenome protein, a weighted count, normalized by the alignable length (the total length of the protein sequence minus the alignment length of the read plus 1) of the protein, was added to that protein entry.
4. If a read has multiple valid alignments to distinct pangenome proteins, the read’s single weighted count was distributed across the corresponding proteins in proportion to the squared percent identity.

The resulting gene family abundances can be summed to generate abundance matrixes for GO, EC, and KO terms with an in-house script. For the GO abundance matrix, merged matrix was additionally filtered to reduce hierarchical redundancy by removing any GO terms that have one or more child terms in the matrix, according to the latest GO annotation (go-basic ontology, release 2022-12-04).

**Methods for batch effect correction**

**ComBat-seq**: With known batch variables and optional biological covariates to be preserved in the adjusted data, ComBat-seq models the batch effect using a negative binomial regression model and maps the original data to the expected batch-free distribution. It assumes a negative binomial distribution for the underlying data to better capture the skewed and over-dispersed nature of integer count data. To adjust the batch effect with ComBat-seq, an unnormalized feature matrix was processed using the combat_seq function from the SVA v3.46.0 ^17^ with default parameters. The resulting batch-adjusted matrix was then subjected to the normalization step.

**LIMMA**: The LIMMA batch correction method fits a linear model to the data and removes the batch-affected component while retaining the covariates of interest. To adjust the batch effect with LIMMA, a normalized feature matrix was processed with using the removeBatchEffect function from the LIMMA v3.54.0 ^18^ with default parameters.

**MMUPHin**: Batch correction function implemented in MMUPHin is a modification of the ComBat framework, accounting for sparse, zero-inflated microbial abundance data. The function disentangles unwanted batch effects while controlling relevant biological covariates by mapping the data into a linear model adjusted for zero-inflation. To adjust the batch effect with MMUPHin, an unnormalized feature matrix was processed with the adjust_batch function from the MMUPHin v1.12.0 ^19^ with default parameters. The resulting batch-adjusted matrix was then subjected to the normalization step.

**ConQuR**: It models the heterogeneous distributions of microbial community profiles through the conditional quantile regression approach, which combines logistic regression and quantile regression models. This non-parametric two-part approach maintains the key nature of microbial read count data, such as zero-inflation, and generates batch-free integer read counts with few restrictions on downstream analyses. To adjust the batch effect with ConQuR, an unnormalized feature matrix was processed with the ConQuR function from the ConQuR v2.0 ^20^ with the following modification to default parameters: simple_match=TRUE. The resulting batch-adjusted matrix was then subjected to the normalization step.

**Naïve batch mean centering**: In this approach, the batch effect was removed from a normalized feature matrix without any assumption of underlying distribution or fitting into a regression model, by adjusting the mean of each feature within a batch to zero.

**Methods for data normalization and transformation**

**Total sum scaling** (TSS): It is a naïve approach that removes bias originating from sequencing depth (library size) by dividing the counts of each feature by the sum of all feature counts, therefore yielding proportional values, *i.e.*, relative abundance.

**Cumulative sum scaling** (CSS): It is a median-like quantile normalization approach that eliminates sequencing depth difference between samples while preserving the variation in total counts between them. Unlike in TSS, CSS prevents the impact of excessively abundant features by calibrating the feature values based on a subset of features, whose value is relatively constant and independent. The method was implemented using the cumNorm function from the metagenomeSeq v1.40.0 ^21^ with the following modification to default parameters: p=0.5.

**Trimmed mean of M-values** (TMM): It is a variance-stabilizing transformation that empirically normalizes the expression level of genes based on the log expression ratios (M values) and the absolute expression levels (A values). The method is originally designed for RNA-seq data and assumes that most features are not differentially abundant. The method was implemented using the calcNormFactors function from the edgeR v3.40.1 ^22^ with the following modification to default parameters: method=“TMM”.

**Centered log-ratio transformation** (CLR): It is a linear transformation that projects proportional data in the n-part Aitchison simplex to n-dimensional Euclidean vector space by performing a log-ratio transformation of relative abundance values to the geometric mean of all feature values. The method was implemented using the clr function from the compositions v2.0-5 with default parameters.

**Isometric log-ratio transformation** (ILR): It is another well-characterized linear transformation that converts proportional data in the n-part Aitchison simplex to (n $-$ 1)-dimensional Euclidean vector space. ILR further decomposes the simplex through an orthonormal basis of the CLR-plane, an inner product space whose vectors are orthogonal to each other, to handle geometric elements in the simplex. The method was implemented using the ilr function from the compositions v2.0-5 with default parameters.

**Arcsine square root transformation** (ARS): It is a variance-stabilizing transformation that expands the skewed binomial distribution of count-derived proportional data to the normal one and reduces heteroskedasticity, *i.e.*, heterogeneity of variance. The method was implemented by sequentially applying the square root and arcsine to the TSS normalized values using the native functions incorporated in R v4.2.3 software.

**Log transformation**: It is a widely used approach for normalizing various types of biological datasets. It is a suitable normalization technique when the distribution is right-skewed and covers a wide range of values since taking a logarithm reduces the effects from outliers and squeezes the distribution to a normal, symmetric shape.

**Handling zero values for log-based normalization**: Any zero values in the feature matrix were replaced by 65% of the minimum non-zero value in the matrix ^23^ before applying CLR, ILR, and LOG.

**Machine learning models for disease classification**

Classification models included in this benchmark were implemented using scikit-learn v1.2.0 ^24^ except for the gradient boosting tree model implemented with xgboost v1.7.2 ^25^. Hyperparameters were selected before model training without any optimization.

**Logistic regression**: It classifies the data into two or more categories by modeling the probability for each class from log-odds, a linear combination of one or more independent variables, using a sigmoid-shaped logistic function. The model is straightforward and understandable as it provides coefficients for each feature, which can be directly interpreted as their importance for classification. Yet, it assumes a linear relationship between features and the log-odds, which might not hold for complex real-world datasets, and is sensitive to outliers that can heavily affect the decision boundary. In our study, the linear variation of the model was implemented using LogisticRegression class with the following modification to default parameters: solver=“sag”, class_weight=“balanced”. Note that L2 (‘ridge’) regularization was applied by default.

**Naïve Bayes**: It is a simple probabilistic classifier built on Bayes’ theorem, where the prefix ‘Naïve’ is for their strong conditional independence assumptions between the features. The over-simplified assumption makes the methods highly scalable and enables them to require a relatively small amount of training data and training time compared to more sophisticated models. However, in the real-world applications, the assumption of independence is rarely satisfied, which may lead to erroneous results. Despite this, the methods are widely used for many practical purposes such as document classification and spam filtering. In our study, the Gaussian Naïve Bayes classifier which assumes the likelihood of the features to be Gaussian, was implemented using GaussianNB class with default parameters.

**Support vector machine** (SVM): It is a non-probabilistic supervised learning model that construct a set of (n $-$ 1)-dimensional hyperplanes that separate given data points lie in n-dimensional space and selects the optimal hyperplane, or the maximum-margin hyperplane, which represents the largest separation between the two classes. The maximum-margin hyperplane is solely determined by data points that lie nearest to it, *i.e.*, support vectors. SVMs are effective in high-dimensional data, especially when they cannot be linearly separated in the original finite-dimensional space. This is due to their ability to establish a non-linear decision boundary by employing the kernel trick, which transforms the data into a higher-dimensional feature space, where the data becomes more amenable to linear separation. In our study, two variations of SVMs were implemented: the linear SVM and the radial basis function (RBF) SVM. The former was implemented using SGDClassifier class with the following modification to default parameters: loss=“modified_huber”, class_weight=“balanced”, and the latter with SVM class with the following modification to default parameters: max_iter=1000, class_weight=“balanced”, probability=True, cache_size=100000.

***k*-nearest neighbors** (kNN): It is a non-parametric supervised learning model that utilizes distance information between samples. It is a variation of instance-based learning that relies on stored information of training samples without generalizing any internal model and classifies previously unseen objects through a simple majority voting of the pre-defined number (*k*) of nearest neighbors of them. The selection of the optimal *k* value is a tradeoff between sensitivity and specificity and is highly data dependent. KNN is also sensitive to the scale of each feature, as it uses the actual distance between instances to classify queries, so the features must be re-scaled to get reliable results. The model was implemented using KNeighborsClassifier class with the following modification to default parameters: weights=“distance”, metric=“manhattan”.

**Decision tree**: It is a non-parametric supervised learning model that infers a set of bifurcating decision rules from the data features. The model constructs a tree graph by recursively splitting the training set in a Boolean manner into subsets based on input features. Leaf nodes in the tree structure represent each class label, and branches represent the combination of features that result in those labels. The decision tree has the advantage of being a white box model, as decision-making processes can be easily explained and visualized. On the flip side, it is susceptible to overfitting and is unstable, as minor changes in the training examples may generate a drastically different tree. The model was implemented using DecisionTreeClassifier class with the following modification to default parameters: class_weight=“balanced”.

**Random forest**: It is an ensemble learning model that combines a large series of independent decision trees generated from samples randomly drawn from the training instances and averages their outputs to return the final prediction. This ‘bagging’ technique compensates for the major weakness of the decision tree, the tendency of overfitting, and decreases the overall variance of the ensembled forest model. While the model generally outperforms a single decision tree, the interpretability is reduced as the decision paths of a multifold of trees are combined. The model was implemented using RandomForestClassifier class with the following modification to default parameters: n_estimators=1000, class_weight=“balanced”.

**Gradient boosting tree**: It is an ensemble learning model based on boosting method, which sequentially combines weak learners, *i.e.*, decision trees, into a single strong learner in the way that each tree learns over the residual of the previous tree and gradually reduces the overall error. The algorithm shares pros and cons with the random forest model but generally performs better ^26, 27^. In our study, extreme gradient boosting (XGB), one of the most widely used variations of gradient boosting trees, was implemented. XGB complements the original algorithm in terms of both model performance and computation efficiency by applying decent regularization techniques and optimized data structure. The model was implemented using XGBClassifier class with the following modification to default parameters: n_estimators=1000. The class weight parameter scale_pos_weight was also set to the proportion of negative and positive labels in the training set.

**Artificial neural network** (ANN): It is a non-linear ML model that approximately emulates the biological neuronal architecture found in animal brains, which consists of interconnected neurons and synapses. The basic units of ANN are artificial neurons that can receive and process input signals and transmit signals that exceed a certain threshold to other neurons. ANN is composed of weighted connections of these artificial neurons, and the training process optimizes the weights to decrease the difference between the prediction of the network and the correct output. In our study, the multilayer perceptron (MLP), which is a subclass of feedforward ANN with fully connected neurons, was implemented using MLPClassifier class with the following modification to default parameters: hidden_layer_sizes=(256,256,256), max_iter=10000, n_iter_no_change=5.

**Supplementary references**

1. Wood DE, Lu J, Langmead B. Improved metagenomic analysis with Kraken 2. Genome Biol 2019; 20:257.

2. Lu J, Breitwieser FP, Thielen P, Salzberg SL. Bracken: estimating species abundance in metagenomics data. PeerJ Computer Science 2017; 3:e104.

3. Ruscheweyh H-J, Milanese A, Paoli L, Karcher N, Clayssen Q, Keller MI, et al. Cultivation-independent genomes greatly expand taxonomic-profiling capabilities of mOTUs across various environments. Microbiome 2022; 10:212.

4. Li H. Aligning sequence reads, clone sequences and assembly contigs with BWA-MEM. arXiv preprint arXiv:13033997 2013.

5. Blanco-Míguez A, Beghini F, Cumbo F, McIver LJ, Thompson KN, Zolfo M, et al. Extending and improving metagenomic taxonomic profiling with uncharacterized species using MetaPhlAn 4. Nat Biotechnol 2023; 41:1633-4.

6. Kim CY, Lee M, Yang S, Kim K, Yong D, Kim HR, et al. Human reference gut microbiome catalog including newly assembled genomes from under-represented Asian metagenomes. Genome Med 2021; 13:134.

7. Parks DH, Chuvochina M, Rinke C, Mussig AJ, Chaumeil P-A, Hugenholtz P. GTDB: an ongoing census of bacterial and archaeal diversity through a phylogenetically consistent, rank normalized and complete genome-based taxonomy. Nucleic Acids Res 2021; 50:D785-D94.

8. Caspi R, Altman T, Billington R, Dreher K, Foerster H, Fulcher CA, et al. The MetaCyc database of metabolic pathways and enzymes and the BioCyc collection of Pathway/Genome Databases. Nucleic Acids Res 2013; 42:D459-D71.

9. Beghini F, McIver LJ, Blanco-Míguez A, Dubois L, Asnicar F, Maharjan S, et al. Integrating taxonomic, functional, and strain-level profiling of diverse microbial communities with bioBakery 3. eLife 2021; 10:e65088.

10. Aleksander SA, Balhoff J, Carbon S, Cherry JM, Drabkin HJ, Ebert D, et al. The Gene Ontology knowledgebase in 2023. Genetics 2023; 224.

11. Ashburner M, Ball CA, Blake JA, Botstein D, Butler H, Cherry JM, et al. Gene Ontology: tool for the unification of biology. Nat Genet 2000; 25:25-9.

12. IUPAC-IUBMB. IUPAC-IUBMB Joint Commission on Biochemical Nomenclature (JCBN) andNomenclature Committee of IUBMB (NC-IUBMB). Eur J Biochem 1999; 264:607-9.

13. Kanehisa M, Sato Y, Kawashima M, Furumichi M, Tanabe M. KEGG as a reference resource for gene and protein annotation. Nucleic Acids Res 2015; 44:D457-D62.

14. Tonkin-Hill G, MacAlasdair N, Ruis C, Weimann A, Horesh G, Lees JA, et al. Producing polished prokaryotic pangenomes with the Panaroo pipeline. Genome Biol 2020; 21:180.

15. Steinegger M, Söding J. MMseqs2 enables sensitive protein sequence searching for the analysis of massive data sets. Nat Biotechnol 2017; 35:1026-8.

16. Cantalapiedra CP, Hernández-Plaza A, Letunic I, Bork P, Huerta-Cepas J. eggNOG-mapper v2: Functional Annotation, Orthology Assignments, and Domain Prediction at the Metagenomic Scale. Mol Biol Evol 2021; 38:5825-9.

17. Leek JT, Johnson WE, Parker HS, Jaffe AE, Storey JD. The sva package for removing batch effects and other unwanted variation in high-throughput experiments. Bioinformatics 2012; 28:882-3.

18. Ritchie ME, Phipson B, Wu D, Hu Y, Law CW, Shi W, et al. limma powers differential expression analyses for RNA-sequencing and microarray studies. Nucleic Acids Res 2015; 43:e47-e.

19. Ma S, Shungin D, Mallick H, Schirmer M, Nguyen LH, Kolde R, et al. Population structure discovery in meta-analyzed microbial communities and inflammatory bowel disease using MMUPHin. Genome Biol 2022; 23:208.

20. Ling W, Lu J, Zhao N, Lulla A, Plantinga AM, Fu W, et al. Batch effects removal for microbiome data via conditional quantile regression. Nat Commun 2022; 13:5418.

21. Paulson JN, Stine OC, Bravo HC, Pop M. Differential abundance analysis for microbial marker-gene surveys. Nat Methods 2013; 10:1200-2.

22. Robinson MD, McCarthy DJ, Smyth GK. edgeR: a Bioconductor package for differential expression analysis of digital gene expression data. Bioinformatics 2009; 26:139-40.

23. Martín-Fernández JA, Barceló-Vidal C, Pawlowsky-Glahn V. Dealing with Zeros and Missing Values in Compositional Data Sets Using Nonparametric Imputation. Math Geol 2003; 35:253-78.

24. Pedregosa F, Varoquaux G, Gramfort A, Michel V, Thirion B, Grisel O, et al. Scikit-learn: Machine Learning in Python. J Mach Learn Res 2011; 12:2825–30.

25. Chen T, Guestrin C. XGBoost: A Scalable Tree Boosting System. Proc 22nd ACM SIGKDD Int Conf Knowl Discov Data Min. San Francisco, California, USA: Association for Computing Machinery, 2016:785–94.

26. Piryonesi SM, El-Diraby TE. Data Analytics in Asset Management: Cost-Effective Prediction of the Pavement Condition Index. J Infrastruct Syst 2020; 26:04019036.

27. Piryonesi SM, El-Diraby TE. Using Machine Learning to Examine Impact of Type of Performance Indicator on Flexible Pavement Deterioration Modeling. J Infrastruct Syst 2021; 27:04021005.
